# High‐fat diet and oral infection induced type 2 diabetes and obesity development under different genetic backgrounds

**DOI:** 10.1002/ame2.12311

**Published:** 2023-04-07

**Authors:** Iqbal M. Lone, Nadav Ben Nun, Aya Ghnaim, Arne S. Schaefer, Yael Houri‐Haddad, Fuad A. Iraqi

**Affiliations:** ^1^ Department of Clinical Microbiology and Immunology, Sackler Faculty of Medicine Tel Aviv University Tel Aviv Israel; ^2^ Department of Periodontology and Synoptic Dentistry, Institute for Dental Craniofacial Sciences Charite‐University of Medicine Berlin Germany; ^3^ Department of Prosthodontics, Dental School The Hebrew University Jerusalem Israel

**Keywords:** collaborative cross, genetic covariance, heritability, high‐fat diet, machine learning, mouse model, obesity, type 2 diabetes

## Abstract

**Background:**

Type 2 diabetes (T2D) is an adult‐onset and obese form of diabetes caused by an interplay between genetic, epigenetic, and environmental components. Here, we have assessed a cohort of 11 genetically different collaborative cross (CC) mouse lines comprised of both sexes for T2D and obesity developments in response to oral infection and high‐fat diet (HFD) challenges.

**Methods:**

Mice were fed with either the HFD or the standard chow diet (control group) for 12 weeks starting at the age of 8 weeks. At week 5 of the experiment, half of the mice of each diet group were infected with *Porphyromonas gingivalis* and *Fusobacterium nucleatum* bacteria strains. Throughout the 12‐week experimental period, body weight (BW) was recorded biweekly, and intraperitoneal glucose tolerance tests were performed at weeks 6 and 12 of the experiment to evaluate the glucose tolerance status of mice.

**Results:**

Statistical analysis has shown the significance of phenotypic variations between the CC lines, which have different genetic backgrounds and sex effects in different experimental groups. The heritability of the studied phenotypes was estimated and ranged between 0.45 and 0.85. We applied machine learning methods to make an early call for T2D and its prognosis. The results showed that classification with random forest could reach the highest accuracy classification (ACC = 0.91) when all the attributes were used.

**Conclusion:**

Using sex, diet, infection status, initial BW, and area under the curve (AUC) at week 6, we could classify the final phenotypes/outcomes at the end stage of the experiment (at 12 weeks).

## Graphical abstract

Type 2 diabetes (T2D) is an adult‐onset and obese form of diabetes caused by an interplay between genetic, epigenetic, and environmental components. Here, we assessed a cohort of 11 genetically different collaborative cross (CC) mouse lines comprised of both sexes for T2D and obesity developments in response to oral infection and high‐fat diet (HFD) challenges. We applied Machine learning (ML) methods to make an early call for T2D and its prognosis. The results showed that classification with random forest could reach the highest accuracy classification (ACC = 0.91) when all the attributes were used. Using sex, diet, infection status, initial body weight, and AUC at week 6, we were able to classify the final phenotypes/outcomes at the end‐stage of the experiment (at 12 weeks).

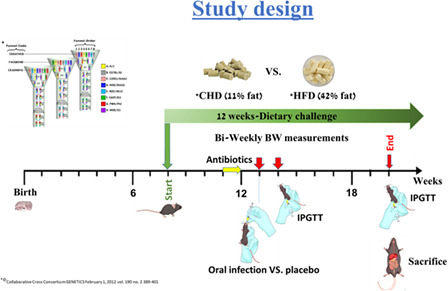

## 1 INTRODUCTION

Mortality due to type 2 diabetes (T2D) is higher in developing countries due to delayed diagnosis and treatment, as T2D is a “silent” disease that develops asymptomatically over the years.^1^ Also with continuous development in living standards, diabetes is increasingly common in the daily lives of people. Therefore, the question of how rapid and accurate diagnosis as well as analysis of diabetes is performed is a topic worth studying. In medicine diabetes is diagnosed based on fasting blood glucose (FBG), glucose tolerance, and random blood glucose levels.^2^, ^3^, ^4^, ^5^, ^6^, ^7^ Obesity, together with age, is the major risk factor for T2D development. Although obesity is not yet a wholly established risk factor for autoimmunity, glucose imbalance and the development of insulin resistance due to an abnormal accumulation of adipose tissue in obese patients correspond with increased events of autoimmune diseases.^8^

Based on the World Health Organization (WHO), the global prevalence of diabetes among adults above age 18 years increased from 4.7% in 1980 to 8.5% in 2014.^9^, ^10^ Furthermore, the growing events of diabetes in middle‐ and low‐income countries were reported to be a major cause of blindness, kidney failure, heart attacks, stroke, and lower‐extremity amputation.^10^ WHO projects that diabetes will be the seventh leading cause of death in 2030.^9^, ^10^ T2D is much more likely to develop among people with metabolic syndrome (METS), which is a collection of risk factors that increase the chance of developing as nonalcoholic fatty liver disease (NAFLD), cardiovascular diseases, stroke, dyslipidemia, kidney failure, and ultimately death from multiple complications.^11^, ^12^ The reports show that overall obesity by body weight (BW), body length (for body mass index calculation), and central obesity can be a strong predictor for T2D development.^13^, ^14^, ^15^ Unfortunately, it is estimated that up to 80% of patients with diabetes will eventually develop METS‐associated diseases.^12^

Furthermore, numerous studies have shown that the prevalence and severity of diabetes‐related complications, including diabetic neuropathy, retinopathy, proteinuria, and cardiovascular complications, are connected.^16^, ^17^ Several studies have reported that patients with diabetes are more prone to developing oral bacterial infections. They are well known to have an impaired defense mechanism and therefore considered to be immunocompromised causing inflammation and influencing T2D development.^18^ The microbiota probably plays an important role in the development of these conditions.^19^ Various studies from our laboratory and collaborators have proven the suitability of the novel and genetically highly diverse mouse genetic reference population, known as collaborative cross (CC), as an appropriate murine model for studying the genetics of complex trait diseases, including diet‐induced T2D.^20^, ^21^, ^22^, ^23^ These studies have shown that genetic background plays a central role in the pathogenesis of T2D, subsequently suggesting genetic factors that may underline this variation in T2D development.^22^, ^23^, ^24^ In the current study, we have assessed the response of the different CC lines for developing T2D and BW gain due to double challenges by high‐fat diet (HFD) and oral infection.

The prior the diagnosis, the much easier it is to control it. Therefore, at this step, machine learning (ML) can help make a preliminary judgment about T2D based on daily physical examination and can serve as a reference for doctors.^25^, ^26^, ^27^ Recently, numerous algorithms have been developed to predict diabetes. This includes traditional ML methods^27^ like support vector machine, decision tree (DT), and logistic regression to deal with large data sets.^28^ In this study, we used a DT and random forest (RF) for prediction. The reason for using ML methods for prediction studies is to make the call before it develops the disease.

Based on these findings, we expanded our current study design by focusing on T2D and obesity developments. It was observed on maintaining different CC lines on HFD challenge and co‐challenge with two oral bacteria under HFD as well as separately.

## 2 MATERIALS AND METHODS

### 2.1 Ethical statement

The Institutional Animal Care and Use Committee (No. 01–19‐013) of Tel Aviv University (TAU) approved all the experimental procedures. These were in line with the Israeli guidelines that follow the National Institutes of Health of USA animal care and use protocols.

### 2.2 CC lines and dietary challenge

The study cohort comprised 471 mice (222 females and 249 males; details of mice are presented in Table 1) produced from the 11 different CC lines provided by the small animal facility at TAU (details of the breeding colony are available at Iraqi et al.^29^). Mice were weaned at age 3 weeks and kept in separate cages by sex and line; they were housed in open‐top cages and maintained in a 12‐h light–dark cycle at a temperature of 21°–23°C with free access to standard rodents' chow diet (CHD) of Altromin 1324 IRR (Altromin Spezialfutter GmbH & Co., Lage, Germany) and water ad libitum. The whole experiment consisted of dietary challenges CHD (as a control group) and HFD provided by TD.88137 (Teklad Global, Harlan Inc, Madison, WI, USA ).

**TABLE 1:** Summary of the used CC lines in our study.

CC line	Diet								Total
	CHD				HFD				
	Noninfection		Infection		Noninfection		Infection		
	Sex								
	♀	♂	♀	♂	♀	♂	♀	♂	
IL72	3	4	4	5	8	5	6	6	41
IL111	2	7	4	5	3	5	4	2	32
IL557	5	3	3	2	2	5	2	3	25
IL711	5	4	5	5	5	5	5	4	38
IL1912	10	12	8	5	9	4	8	14	70
IL3348	4	3	3	6	3	5	5	7	36
IL3912	6	13	4	9	5	7	13	8	65
IL4141	4	8	7	3	4	4	8	5	43
IL5001	4	4	3	5	7	7	–^a^	2	32
IL5003	2	2	3	4	2	4	6	3	26
IL6018	4	4	11	8	8	12	5	11	63
Total	49	64	55	57	56	63	62	65	471

*Note*: The name of each CC line is designated as IL#, which appears under the column CC lines. Number of males and females in each experimental group, including CHD noninfection, CHD infection, HFD noninfection, and HFD infection, is provided.

Abbreviations: CC, collaborative cross; HFD, high‐fat diet.

^a^ The group of females from IL5001 is missing due to unavailability of female mice in the animal facility (IL5001 extinct from TAU colony).

### 2.3 Study design

The experimental period spanned 12 weeks with two environmental challenges of HFD and oral infection with mixed‐oral bacteria. At the initial time point (8‐week‐old mice), BW was measured using an electronic balance. The mice were divided into two dietary groups, in which HFD (42% fat) was provided for the experimental group and CHD (11% fat) for the control group. At week 5 of the experiment (age 13 weeks), mice from both dietary conditions were further divided into two groups for the infection challenge, where the experimental groups were orally infected with mixed‐oral bacteria by gavage and control groups were placebo‐infected without bacteria as a control group. At week 12 of the experiment, glucose tolerance ability was assessed using the intraperitoneal glucose tolerance test (IPGTT). After overnight recovery, mice were weighed and killed. The four experimental groups were (1) CHD/noninfected, (2) CHD/infected, (3) HFD/noninfected, and (4) HFD/infected.

### 2.4 IP glucose tolerance test and area under the curve calculation

This test was performed to detect disturbances in glucose metabolism that can be related to diabetes or prediabetic conditions elaborated in our previous publications.^23^, ^24^

### 2.5 Bacterial cultivation

*Porphyromonas gingivalis* (*Pg*) strain ATCC 33277 and *Fusobacterium nucleatum* (*Fn*) strain PK 1594 were grown in peptone yeast extract containing hemin and vitamin K (Wilkins Chalgren broth, Oxoid Ltd, Lenexa, KS, USA) in an anaerobic chamber with 85% N_2_, 5% H_2_, and 10% CO_2_. The contents were washed thrice in 1% phosphate‐buffered saline (PBS). A spectrophotometer was used to measure the bacterial concentration at an OD of 650 nm = 0.1 for Pg, corresponding to 1010 bacteria per milliliter; and OD 660 nm = 0.26 for *Fn*, to 109 bacteria per milliliter. A confocal microscope was used to check the quality control to eliminate contamination.

### 2.6 Oral infection challenge

The mice were treated with antibiotics to standardize the oral microbiota status before the infection. The use of sulfamethoxazole (10 mL/500 mL) in water administration for 10 days, followed by 3 days of recovery (antibiotic‐free). Then the infection challenge was started with oral infection of 400 μL of the mixed‐oral bacteria (*Pg* and *Fn*) per mouse. The infection procedure was repeated every other day thrice for 5 days of week 5. In parallel, the treatment of placebo infection to the control groups with 400 μL of 2% Carboxymethyl cellulose (CMC) in distilled water and 1% PBS (CMC–PBS, 2:1) was given.

### 2.7 Data analysis

The IBM SPSS (Statistical Package for the Social Sciences) software platform (version 24) was used for data analysis. The variation between the CC lines and the significance (*p* < 0.05) was assessed using one‐way analysis of variance (ANOVA).

### 2.8 Heritability and genetic coefficient of variation analysis in the CC lines

The heritability estimates were obtained from unpublished data of a wide variety of traits presently being studied at TAU on the same lines as of Iraqi et al.^30^ Further studies referred were Garcia‐Gonzalez et al^31^ and Houle.^32^

### 2.9 Computational methods

#### 2.9.1 Classification models

In this study, we used DT, naive Bayes (NaBa), k‐nearest neighbors (KNN), and RF as the classifiers. All classifiers were implemented using the Scikit‐Learn Python package.

#### 2.9.2 Decision tree

The DT model with a tree structure can describe the process of classification instances based on features.^33^ In this study, we used Scikit‐Learn's default implementation, with a maximal tree depth of 10.

#### 2.9.3 Naive Bayes

The model of NaBa classification is based on a Bayesian network (BN) structure. A BN refers to a graphical model for probability associations between a set of variables. In this study, we used Scikit‐Learn's default Gaussian NaBa.

#### 2.9.4 k‐nearest neighbors

In the KNN technique, the nearest neighbor is measured with respect to the value of k, which defines how many nearest neighbors need to be examined to describe the class of a sample point. In this study, we used Scikit‐Learn's default implementation for K‐neighbors classifier (five neighbors).

#### 2.9.5 Random forest

This algorithm was proposed by Breiman^34^, ^35^ as a multifunctional ML method performing the tasks of prediction and regression. In addition, RF is based on bagging, and it plays an important role in ensemble ML.^35^, ^36^ It has been employed in several biomedical research studies.^37^, ^38^ In this study, we used Scikit‐Learn's default implementation, with 100 trees in a forest.

#### 2.9.6 Regression models

We used two regression models, namely linear regression and KNN regression, as they produced some meaningful results for our data.

#### 2.9.7 Model validation

To evaluate the ability of the model, usually two validation methods, namely hold‐out method and k‐fold cross‐validation method, are used.^39^, ^40^, ^41^, ^42^ Based on the goal of each problem and the size of data, we can choose a method of choice to solve the problem.^43^ In this study, we used K (4)‐fold cross‐validation.

## 3 RESULTS

Based on ANOVA, a significant sex effect of BW was found among the 11 different CC lines. Males were found to be heavier and bigger than females.

### 3.1 Sex effects within the CC line vary between different lines at different time points

The sex effects for BW and glucose tolerance ability, at weeks 6 and 12, in response to dietary and infection challenges, varied between different lines. In all the groups studied, for all the CC lines, males showed higher BW (g) values than females (Figures S1 and S2, Supplementary Material available at https://tinyurl.com/43ck5ekh), except IL5003 and IL3912 females that showed a decline in BW throughout the first 6 weeks of the experiment and then from weeks 6 to 12 showed an increase in BW (Figure S1A,C, Supplementary Material available at https://tinyurl.com/43ck5ekh), respectively. In the case of IL3348 and IL5003 males, a decline in BW was observed from weeks 6 to 12 on HFD/without infection (Figure S2C, Supplementary Material available at https://tinyurl.com/43ck5ekh).

**FIGURE 1:**
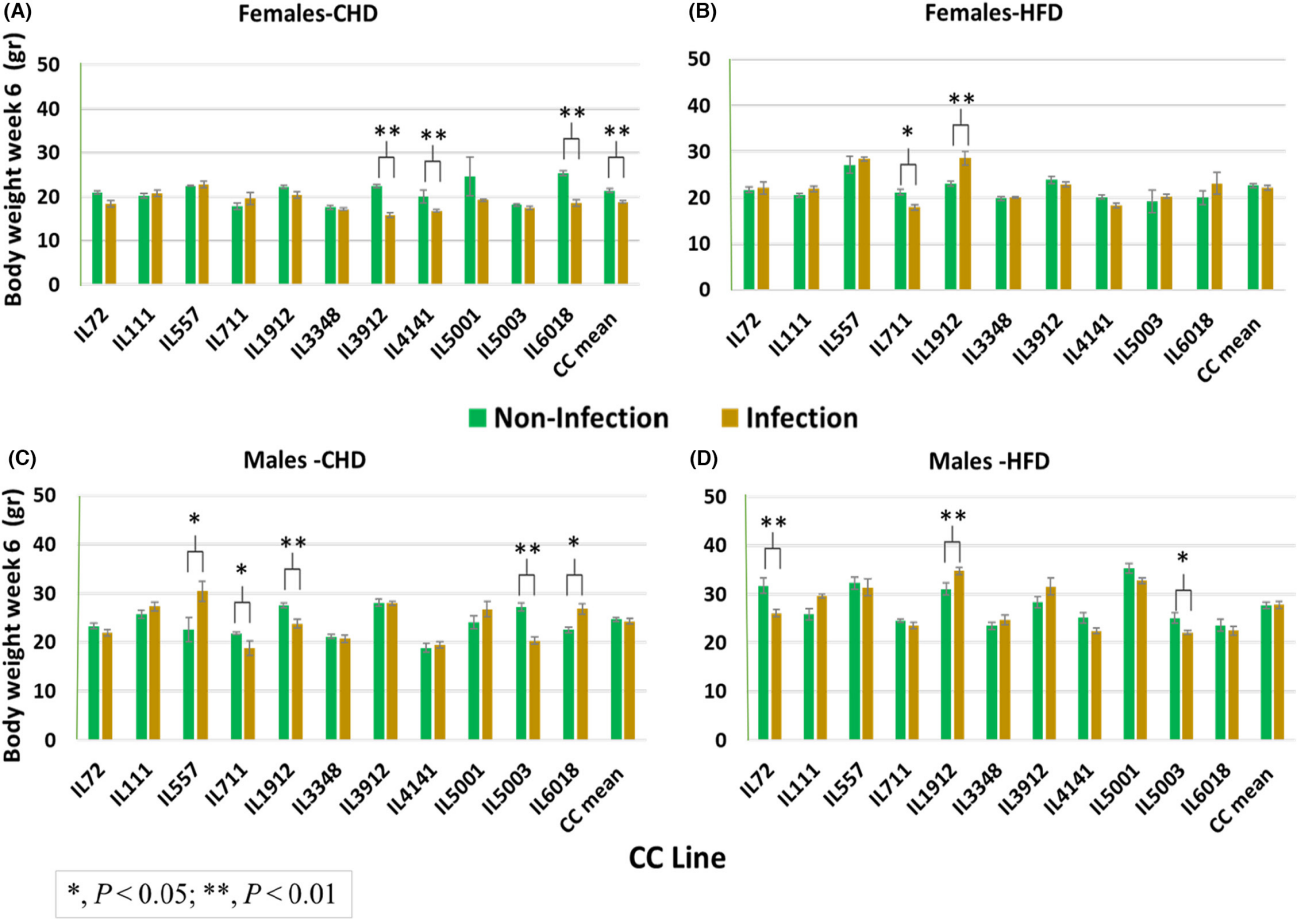
Week 6‐time point BW (body weight, in grams) of 11 CC (collaborative cross) lines separately for females and males, after 6 weeks on CHD compared with the same lines after 6 weeks on HFD (high‐fat diet) while infected and noninfected status as presented. (A and B) Week 6 BW means (±SE [standard error]) for CHD and HFD of females, respectively. (C and D) Week 6 BW means (±SE) for CHD and HFD of males, respectively, by lines. The *x‐*axis represents the different CC lines; the *y*‐axis represents BW (g) at week 6 of the experiment.

**FIGURE 2:**
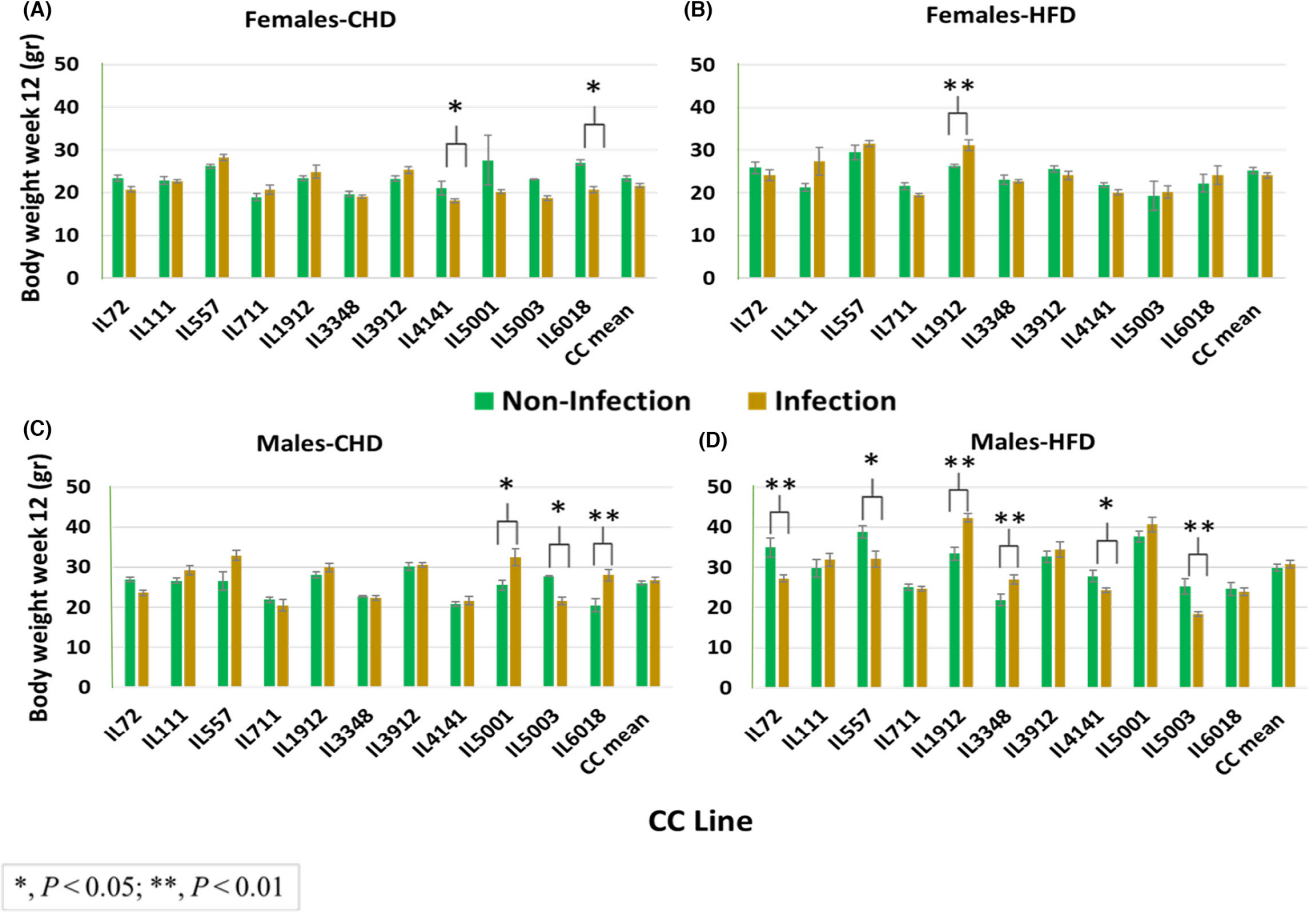
End time BW (body weight, in grams) of 11 CC (collaborative cross) lines separately for females and males, after 12 weeks on CHD and on HFD (high‐fat diet) while infected and noninfected status as presented. (A and B) The BW means (±SE [standard error]) at the end time point for CHD and HFD of females, respectively, by lines. (C and D) End time point BW means (±SE) for CHD and HFD of males, respectively, by lines. The *x*‐axis represents the different CC lines; the *y*‐axis represents BW (g) at the end time point of the experiment (week 12).

The BW profile at week 6 varied between and within the different CC lines in both sexes. Overall, as shown in Figure 1A–D, after 6 weeks of the dietary challenge and infection condition, significant (*p* < 0.001) changes were observed between females on CHD noninfection and CHD infection (Figure 1A). Females on CHD without infection show higher BW (g) than females on CHD with infection. The BW at week 12 also varied between the different CC lines with a sex effect. The overall situation after a 12‐week period of nutritional challenge and infection state is shown in Figure 2A–D.

**FIGURE 3:**
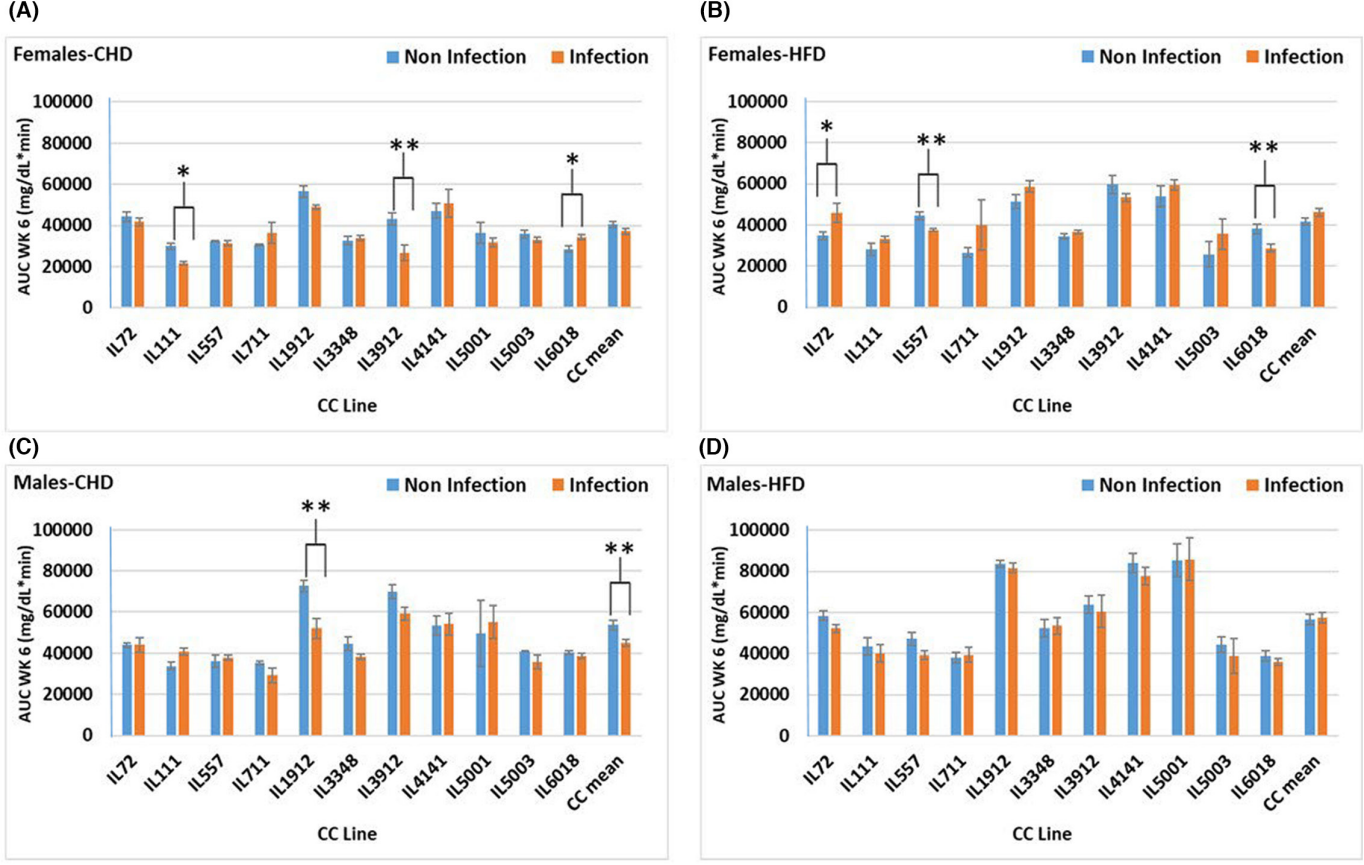
Blood glucose levels (mg/dl) during intraperitoneal glucose tolerance test (IPGTT) represented here as AUC (area under the curve) of 11 CC (collaborative cross) lines separately for females and males, after 6 weeks on CHD or on HFD (high‐fat diet) while infected and noninfected status as presented. (A and B) Means (±SE [standard error]) of the total AUC on CHD and on HFD for females; (C and D) Means (±SE) of total AUC on CHD and on HFD for males. The *x*‐axis represents the different CC lines; the *y*‐axis represents total AUC (mg/dl min) at week 6.

**FIGURE 4:**
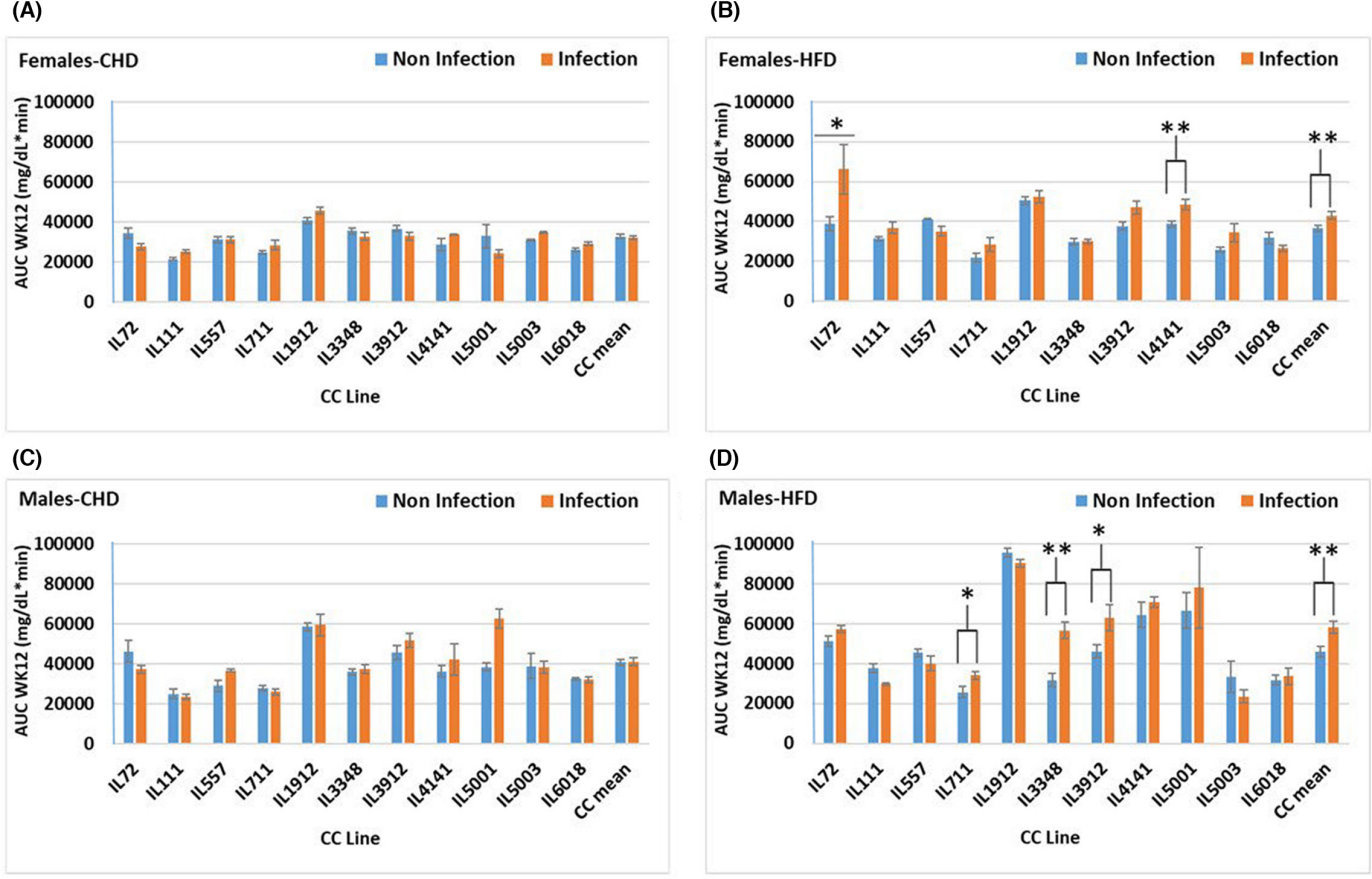
Blood glucose levels (mg/dl) during intraperitoneal glucose tolerance test (IPGTT) represented here as AUC (area under the curve) of 11 CC (collaborative cross) lines separately for females and males, after 12 weeks on CHD or on HFD (high‐fat diet) while infected and noninfected status as presented. (A and B) Means (±SE [standard error]) of the total AUC on CHD and on HFD for females; (C and D) Means (±SE) of total AUC on CHD and on HFD for males. The *x*‐axis represents the different CC lines; the *y*‐axis represents total AUC (mg/dl min) at week 12.

The overall results observed in the area under the curve (AUC, min × mg/dl) were coherent with BW results presented in Figures 3 and 4. In the case of females the overall AUC in the CHD challenge, noninfectious versus infectious, was higher even at week 6 (Figure 3A). In males on HFD infection/noninfection (Figure 3D), only a slight increase was observed in favor of the infected group. Sex effects revealed a highly significant (*p* = 0.000) AUC in all the groups even at each time point. At week 12 on HFD challenge (Figure 4B), the total AUC was observed to be significantly higher (*p* < 0.01) in the infected group than in the noninfected group, except IL6018 showing similar response under reverse conditions in the noninfected versus the infected groups.

Figure S3 shows that the BW differences between males and females within a line were greater on HFD for the noninfected groups. In the case of females on CHD challenge (Figure S3A, Supplementary Material available at https://tinyurl.com/43ck5ekh), significant BW changes were observed between the noninfected and infected groups in IL72, although the noninfected group had a higher BW gain; and in the case of females on HFD (Figure S3B, Supplementary Material available at https://tinyurl.com/43ck5ekh), an overall significant (*p* < 0.05) change in percentage BW gain was observed between the noninfected and the infected groups, where the infected group had a higher percentage of BW gain. In addition, females on HFD challenge had reached a higher percentage of BW gain compared to CHD challenge in both noninfected and infected conditions. Significant differences were observed in IL1912 and IL4141.

Regarding males on CHD challenge (Figure S3C, Supplementary Material available at https://tinyurl.com/43ck5ekh), overall, the infected group had reached a higher percentage of BW gain. The most significant changes observed between the noninfected and infected groups were in IL1912, IL5003, and IL6018 at which the infected group had a higher percentage of BW gain during the experiment than the noninfected group: IL1912 (*p* < 0.05) and IL5003 (*p* < 0.05). IL6018 (*p* < 0.01) in the noninfected group showed a decline in BW.

For males on HFD challenge (Figure S3D, Supplementary Material available at https://tinyurl.com/43ck5ekh), the infected group had reached a higher percentage of BW gain than the noninfected group. Also, males on HFD had reached a higher percentage of BW gain in both infection conditions, infected versus noninfected groups. The most significant changes observed between the noninfected and infected groups were shown with lines; IL557, IL1912, and IL5003. IL557 and IL5003. in an interesting manner IL5003 showed different results between the two diets (Figure S3C,D, Supplementary Material available at https://tinyurl.com/43ck5ekh).

### 3.2 Variations in glucose tolerance ability in response to dietary and infection challenges in different CC lines at different time points

The ΔAUC_6–12_ (mg/dl min) values varied between the different CC lines for both sexes, in both dietary and infection challenge conditions. Changes in glucose tolerance ability by age varied between the CC lines. The general direction of change was toward a decrease in AUC (mg/dl min) levels with age, indicating an improvement in glucose tolerance ability in response to glucose load despite the infection condition even with the case of male mice under HFD with infection (Figure S4D, Supplementary Material available at https://tinyurl.com/43ck5ekh). Yet changes with age varied between the CC lines and sexes, leading to opposite responses. Thus, some CC lines showed a decrease, whereas others showed an increase.

For females under CHD challenge (Figure S4A, Supplementary Material available at https://tinyurl.com/43ck5ekh), the overall ΔAUC_6–12_ values for both infection conditions show an improvement in glucose tolerance ability; the improvement is higher in the noninfected group than in the infected group, which is expressed by a higher absolute value of ΔAUC. In contrast to the mean ΔAUC of the overall population for the noninfected group, IL3348 showed the highest ΔAUC_6–12_, indicating slow glucose tolerance ability.

Regarding the infected group, three CC lines IL111, IL3348, and IL5003 showed a positive ΔAUC_6–12_, indicating a week glucose tolerance ability, presented in Figure S4A, and the most significant deterioration occurred in IL3912. For females on HFD challenge (Figure S4B, Supplementary Material available at https://tinyurl.com/43ck5ekh), both infection conditions showed an improvement in the overall ΔAUC_6–12_ values.

In contrast to the mean of the overall population for the noninfected group, IL72 and IL111 showed the highest ΔAUC_6–12_ values, indicating a weak glucose tolerance ability. Regarding the infected group, two CC lines, IL72 and IL111, showed a positive ΔAUC_6–12_ value, indicating a weak glucose tolerance ability (Figure S4B, Supplementary Material available at https://tinyurl.com/43ck5ekh); IL72 showed the most significant deterioration.

For males on CHD challenge (Figure S4C), overall, both infection conditions showed an improvement in glucose tolerance ability. The improvement was higher in the noninfected group than in the infected group, expressed by a higher absolute ΔAUC.

In contrast to the mean of the overall population for the noninfected group, IL72 showed the highest ΔAUC_6–12_ value, indicating slower glucose tolerance ability. Regarding the infected group, in contrast to the mean of the overall population, three CC lines, IL1912, IL5001, and IL 5003, showed a positive ΔAUC_6–12_ value, indicating worsening in glucose tolerance ability, (Figure S4C, Supplementary Material available at https://tinyurl.com/43ck5ekh).

For males on HFD challenge (Figure S4D, Supplementary Material available at https://tinyurl.com/43ck5ekh), overall, the noninfected group showed an improvement in glucose tolerance ability. In contrast to the mean of the overall population for the noninfected group, IL1912 showed the highest ΔAUC_6‐12_ value, indicating slower glucose tolerance ability.

Contrary to that discussed thus far, overall, for the infected group the direction was toward worsening glucose tolerance ability, showing a positive ΔAUC_6–12_ value. Aggravation appears mainly in the following lines: IL72, IL1912, IL3348, and IL 3912 (Figure S4D, Supplementary Material available at https://tinyurl.com/43ck5ekh).

Interestingly, IL72 on HFD with infection co‐challenge showed worsening in glucose tolerance ability with a positive ΔAUC_6–12_ value in both female mice (Figure S4B, Supplementary Material available at https://tinyurl.com/43ck5ekh) and male mice (Figure S4D, Supplementary Material available at https://tinyurl.com/43ck5ekh), with 20 430 and 5151 mg/dL min, respectively, indicating worsening in females was more intense.

Moreover, among male mice in both diet challenges under infection condition, IL1912 showed that glucose tolerance ability was worsening, which was reflected in the positive ΔAUC_6–12_ value (Figure S4C,D, Supplementary Material available at https://tinyurl.com/43ck5ekh). On HFD challenge (Figure S4D, Supplementary Material available at https://tinyurl.com/43ck5ekh) males of IL1912 showed a deterioration in glucose tolerance ability also in the noninfection condition, which has a positive ΔAUC_6–12_ value.

### 3.3 Variations in FBG in response to dietary and infection challenges in different CC lines at different time points

Our results have shown variations in FBG levels for females at week 6 (Figure S5A, Supplementary Material available at https://tinyurl.com/43ck5ekh) between the different CC lines, in addition to variations within a line comparing the four experimental groups.

Overall, the highest value of FBG was observed in the CHD group without infection, which differs significantly (*p* < 0.01) compared to infection condition on CHD. Overall, females on HFD showed a similar pattern to the CHD though the highest values were observed in the noninfected group. Focusing on diet effect under the infection condition, we observed a significant (*p* < 0.05) difference, which is translated by a higher FBG in the HFD group with infection versus CHD group with infection.

Regarding females at week 12 (Figure S5B, Supplementary Material available at https://tinyurl.com/43ck5ekh), overall, lowest values of FBG were observed in all experimental groups. Accordingly comparing to week 6 and significant (*p* < 0.01) differences were observed between the infection conditions on HFD, noninfection group has higher value of FBG versus the infection group.

Focusing on diet effect in the noninfection condition, we observed a significant (*p* < 0.01) difference translated by a higher FBG in the HFD group without infection versus CHD without infection.

For males at week 6 (Figure S5C, Supplementary Material available at https://tinyurl.com/43ck5ekh), the highest FBG values were observed in the HFD group with infection, with significant (*p* < 0.05) difference between the infection conditions on CHD, though the noninfected group had a higher FBG value than the infected group.

Focusing on diet effect in the infection condition, we observed a significant (*p* < 0.01) difference translated by a higher FBG in the HFD group with infection than in the CHD group with infection. Compared to females, males showed higher FBG values in all experimental groups at week 6 (Figure S5A,C, Supplementary Material available at https://tinyurl.com/43ck5ekh).

As in females, males also showed the lowest FBG values in all experimental groups at week 12 compared to week 6; in addition, the highest FBG value in males was observed in the HFD group without infection.

A significant difference was observed at week 6 as well as week 12, among infection conditions on CHD and on diet effect under infection condition, that is, infected group (Figure S5D, Supplementary Material available at https://tinyurl.com/43ck5ekh). In addition, a significant (*p* < 0.05) difference was observed between the infection conditions on CHD at which the noninfected group had a higher FBG value than the infected group.

Focusing on diet effect under the infection condition, significant (*p* < 0.05) differences were observed among the different groups, which were translated by a higher FBG in the HFD with infection than the CHD with infection.

### 3.4 Pearson's correlation between BW and glucose tolerance

**FIGURE 5:**
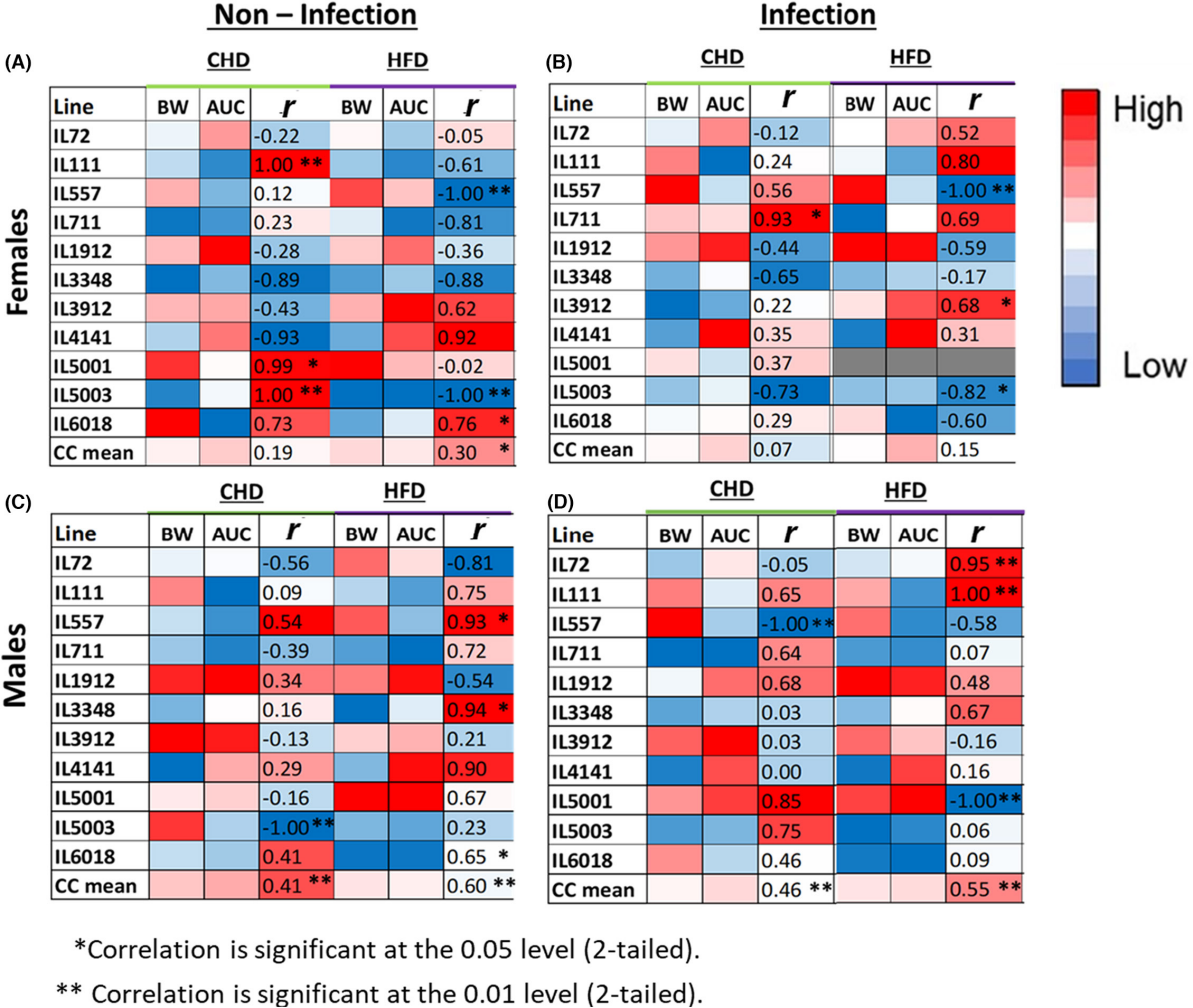
Heat map showing Pearson's correlation coefficient for two traits: BW (body weight) and glucose tolerance referred here as AUC (area under the curve), at week 6 of the experiment for (A and B) females and (C and D) males, among the four different conditions of the experiment. Part figures A and C present the noninfection condition, and B and D present the infection condition. Each map presents both dietary challenges, CHD and HFD (high‐fat diet). Based on the color key, the correlation coefficient −1 ≤ *r* ≤ 1 is significant at **p < 0.01 and *p < 0.05.

Variations were observed between different CC lines and within the same line when comparing diets and infection conditions. For females in the noninfected group (Figure 5A), overall, a weak positive correlation was observed between the two traits on both dietary challenges, with a value of *r* = 0.19 on CHD and *r* = 0.30 on HFD. On the CHD challenge three CC lines, IL111, IL5001, and IL5003, showed a significant (*p* < 0.05) positive correlation between the two traits (BW week 6 and AUC week 6). On the HFD challenge under the noninfection condition, three CC lines, IL3912, IL4141, and IL6018, showed a positive correlation between the two traits.

For females in the infected group (Figure 5B), on the CHD challenge, a significant (*p* < 0.05) positive correlation was observed in IL711 (Figure 5A). Unlike the significant (*p* < 0.05) positive correlation observed in IL5003 under the noninfection condition on CHD (Table 3), negative correlation was observed under the infection condition within the same diet (CHD).

For males in the noninfected group (Figure 5C) as well as in the infected group (Figure 5D), overall, a positive correlation was observed between the two traits on both dietary challenges. For males in the infected group on HFD (Figure 5D), a significant (*p* < 0.01) positive correlation was observed in IL72 and IL111. IL72 showed a high positive correlation (*r* = 0.95), whereas in all other cases in the same line among males, a negative correlation was observed between the two traits.

**FIGURE 6:**
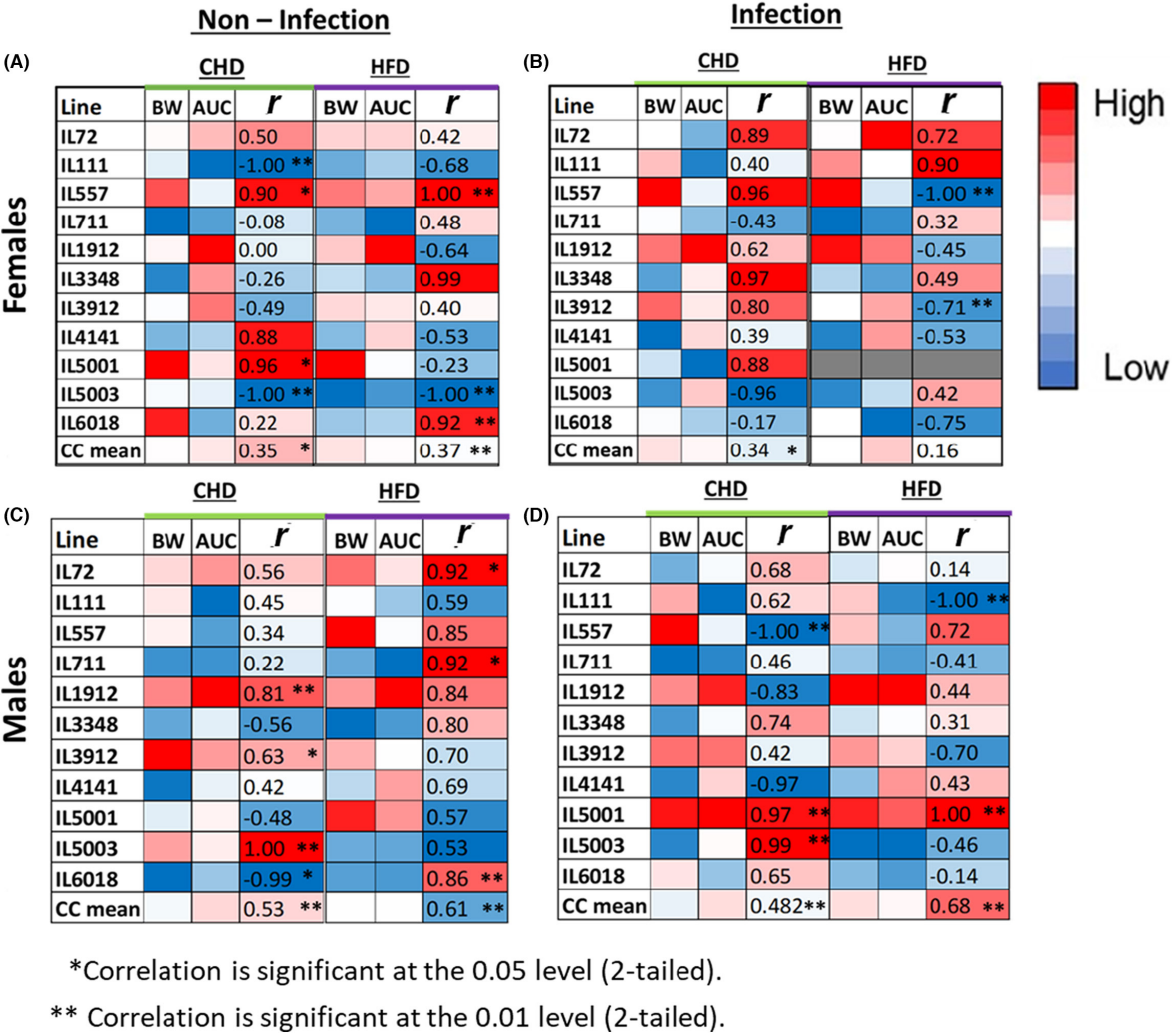
Heat map showing Pearson's correlation coefficient for two traits: BW (body weight) and glucose tolerance referred here as AUC (area under the curve) at week 12 of the experiment for (A and B) females and (C and D) males, among the four different conditions of the experiment. Part figures A and C present the noninfection condition, and B and D present the infection condition. Each map presents both dietary challenges, CHD and HFD (high‐fat diet). According to the color key, correlation coefficient −1 ≤ *r* ≤ 1 is significant at **p < 0.01 and *p < 0.05.

Variations were observed between the lines and between the same CC line when comparing diets and infection conditions. For females in the noninfected group (Figure 6A) as well as the infected group (Figure 6B), overall, a mixed positive correlation was observed between the two traits on both dietary challenges of CHD and HFD. On the HFD challenge, high variations were observed between the CC lines in terms of the correlation between the traits, yet the most notable was observed in IL557 and IL3912, which showed opposite correlation on CHD in the infected group.

For males in the infected group (Figure 6D), overall, a positive correlation was observed on both dietary challenges. Two CC lines, IL5001 and IL5003, showed a significant (*p* < 0.01) positive correlation between the two traits. Three CC lines, namely IL557, IL1912, and IL4141, showed a highly negative correlation between the traits than they showed with different directions of correlation in the noninfected group within the same diet (Figure 6C).

For males in the infected group on HFD (Figure 6D), a significant (*p* < 0.01) positive correlation was observed in IL5001, with the same direction of correlation on CHD in the infected group. The highest negative correlation was observed in IL111, whereas in all other cases in the same line among males, a positive correlation was observed between the two traits. The heat maps of all the individual CC lines are shown in Figures S6–S31.

### 3.5 Heritability and genetic coefficient of variation

Table 2 presents heritability and genetic coefficient of variation values for a variety of the traits studied. The heritability values of these traits are generally in the range of 0.45–0.85, whereas the CV_G_ value has been observed to be in the range of 0.08–0.40, much higher than the benchmark of 0.071. Thus, the data show that an absolute magnitude of genetic variation among the CC lines observed is higher than that found within a typical outcrossing population.

**TABLE 2:** Summary of heritability and genetic coefficient of variation in the used CC lines in our study.

Sex	Trait	H2				CV_G_			
		CHD		HFD		CHD		HFD	
		Noninfection	Infection	Noninfection	Infection	Noninfection	Infection	Noninfection	Infection
♀	BW_6_	0.780	0.454	0.582	0.623	0.111	0.089	0.137	0.154
	BW_12_	0.691	0.454	0.582	0.565	0.105	0.131	0.165	0.162
	AUC_6_	0.720	0.523	0.636	0.461	0.255	0.231	0.254	0.230
	AUC_12_	0.578	0.800	0.567	0.459	0.174	0.203	0.220	0.270
♂	BW_6_	0.703	0.721	0.669	0.772	0.136	0.153	0.153	0.189
	BW_12_	0.662	0.718	0.650	0.850	0.137	0.164	0.192	0.263
	AUC_6_	0.631	0.546	0.751	0.750	0.289	0.214	0.330	0.332
	AUC_12_	0.648	0.723	0.747	0.812	0.276	0.312	0.153	0.402

*Note*: Each experimental group comprises CHD noninfection, CHD infection, HFD noninfection, and HFD infection.

Abbreviations: AUC, area under the curve; BW, body weight; CV_G_, genetic coefficient of variation; HFD, high‐fat diet.

### 3.6 Classification and regression results

For better comparison, first, we used all features for predicting different aspects of diabetes, and the results are presented in Tables 3 and 4. Tables 3 and 4 show that different classification algorithms perform differently for the same line. In Table 3, for line 5001 DT and RF performed well, with scores of 0.866 and 0.933, respectively. However, for the same line NaBa and KNN did not perform as well, with respective receiver operating characteristic (ROC) AUCs of 0.73 and 0.58. In Table 4 we were able to observe that for line 3912 NaBa and RF performed well with 0.832 and 0.905 ROC AUCs, respectively, whereas DT and KNN had significantly lower scores of 0.7 and 0.466, respectively.

**TABLE 3:** Classification results.

Model/line	3912	72	1912	5001	5003	4141	111	6018	3348	557	711
*N*	65	41	70	32	26	43	32	63	36	25	38
Decision trees	0.553	0.583	0.823	**0.866**	0.55	0.785	0.607	0.654	0.724	0.758	0.528
Naive Bayes	0.574	0.632	**0.954**	0.73	0.409	**0.913**	0.61	0.746	**0.911**	**0.856**	0.576
k‐Nearest neighbors	0.388	0.408	**0.903**	0.58	0.773	**0.888**	0.69	0.804	0.524	**0.846**	0.503
Random forest	0.581	0.553	**0.961**	**0.933**	0.533	**0.93**	0.679	0.751	**0.913**	**0.887**	0.662

*Note*: The input features are sex, diet, infection, initial body weight, and AUC at week 6. The output features are classification of AUC at week 12—larger/smaller than the 80th percentile of the data. Using the sex, diet, infection, initial body weight, and AUC‐6 to classify whether AUC‐12 would be in the top 20% of the data has produced high AUC values for most lines. Bold indicates statistical significant value (*p* < 0.05).

Abbreviation: AUC, area under the curve.

**TABLE 4:** Classifying the final body weight (80th percentile as well) has produced very high AUC values.

Model/line	3912	72	1912	5001	5003	4141	111	6018	3348	557	711
*N*	65	41	70	32	26	43	32	63	36	25	38
Decision trees	0.7	0.694	**0.99**	0.527	0.68	0.754	0.643	0.688	0.768	0.768	0.773
Naive Bayes	**0.832**	0.767	**1**	0.686	**0.817**	**0.875**	0.688	**0.831**	**0.826**	**0.85**	**0.861**
k‐Nearest neighbors	0.466	0.625	**0.832**	0.69	0.775	0.733	0.751	0.466	0.658	0.831	0.654
Random forest	**0.905**	**0.83**	**1**	0.716	**0.886**	**0.933**	0.66	**0.89**	**0.874**	**0.902**	**0.93**

*Note*: All values are averages of a fourfold cross‐validation over 10 iterations. Bold indicates statistical significant value (*p* < 0.05).

Furthermore, the same algorithm performed differently for each line. As observed in Table 3 the results for KNN vary from 0.388 in line 3912 to 0.903 in line 1912. In Table 4, NaBa varies from 0.686 in line 5001 to a perfect one in line 1912.

In addition, RF has the overall best result among the four classifiers, as it exceeds the 0.85 ROC AUC mark for the maximum number of CC lines. Table 4 shows that RF was able to classify with a high ROC AUC for all the lines except 5001 and 111, for which no algorithm was able to classify with a high success rate. Table 4 shows that in the final BW RF performed better than the others, and the ROC AUC is above 0.80 in most lines. Based on the matrix, we chose the first five features, which are sex, diet, infection, initial BW, and AUC‐6, to predict AUC‐12 for all the CC lines under study, and the results are presented in Tables 3 and 4. In the data set, this method, which used RF as the classifier, performed the best. Tables 3 and 4 show that RF is able to predict better diabetes severity.

**FIGURE 7:**
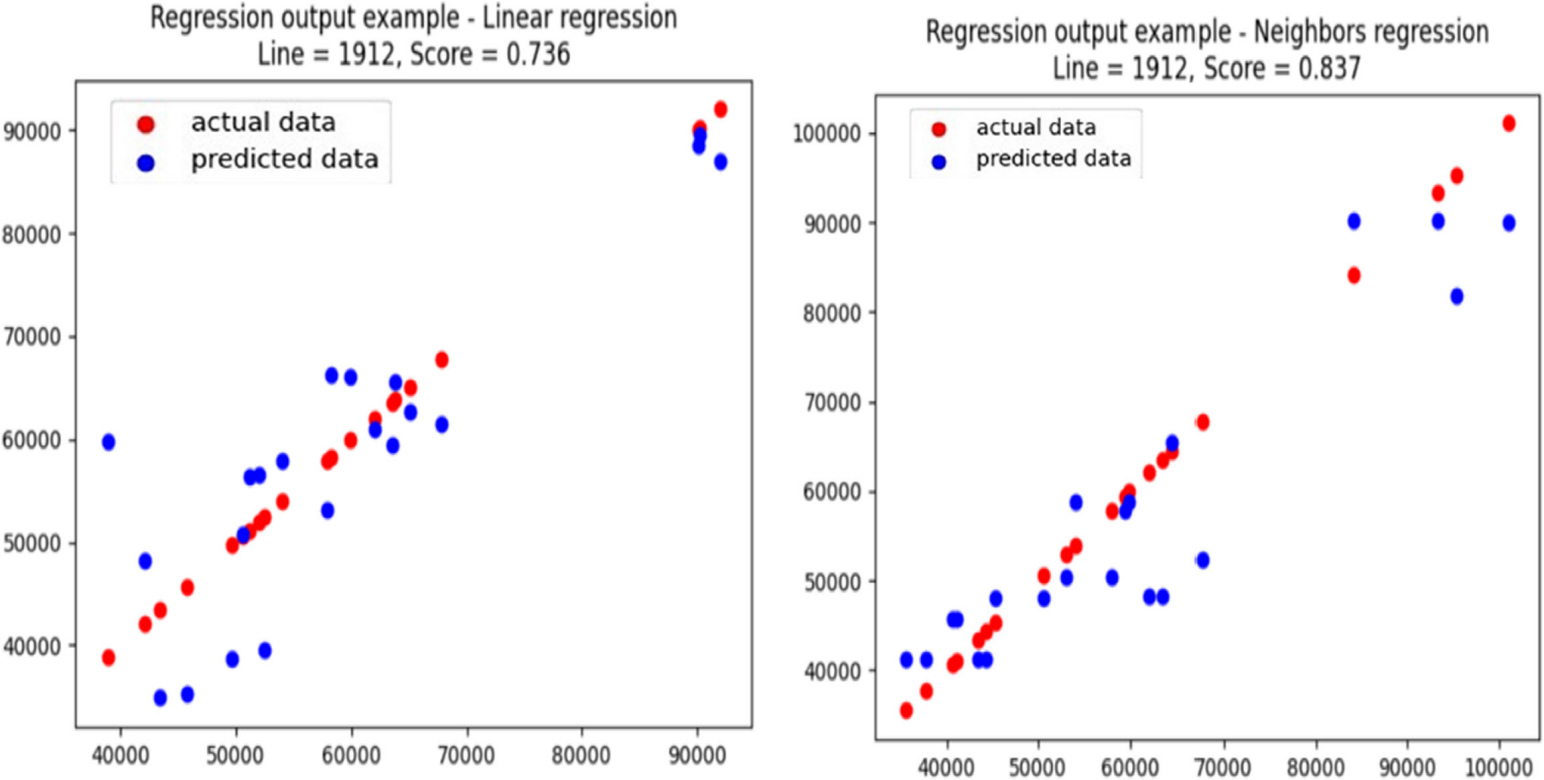
The KNN regression results of CC (collaborative cross) line 1912 for AUC‐12 (area under the curve) performed on the entire data. The predicted score is very high, that is, 0.83. Both the actual (red) and predicted (blue) values can be observed in the figure. The linear regression results were the average results of 100 iterations, with a test set of 40%. Here we observed that also for *R* ^2^ = 0.73 the majority is predicted very well.

Figure 7 shows that regression may be suitable for some CC lines but not for all the lines under study, which clearly demonstrates the accuracy to make a better comparison. Figure 7 shows an example of a successful regression, as the *sss* values are greater than 0.7. We also observed a difference in the performance of the two algorithms, as neighbors regression produced an average *R* ^2^ of 0.837, whereas linear regression produced an average *R* ^2^ of 0.736.

## 4 DISCUSSION

Obesity is a major public health problem and a risk factor for several chronic diseases, including T2D.^44^, ^45^ In China, diabetes reached epidemic dimensions in adult population during 2007 and 2008, demonstrating a higher prevalence among males compared to females.^46^, ^47^ On the contrary, in southern Africa, diabetes prevalence in females was higher than the global average but about the same in males.^48^ These findings confirm the important role that the host genetic background plays in disease development, as also revealed by Kwok et al.^49^ Our results demonstrated a high contribution of host genetic background and sex in response to infection, diet, and their interactions in cohesion with an earlier study from our laboratory by Karkar et al.^50^

Regarding blood glucose tolerance ability, males showed higher AUC on both dietary challenges and infection conditions than females in the two time points of IPGTT performance (weeks 6 and 12), as also observed by Atamni et al.^23^ A possible interpretation might be the protective effect of the female sex hormone, estrogen.^51^ However, these findings varied at the level of individual CC lines whereby few lines presented an opposite effect (females responded more than males).

Regarding AUC in the early phase after infection (week 6), in both sexes, on CHD, higher blood glucose level was observed in the noninfected group than in the infected group, also proposed by Milhem et al,^52^ whereas on HFD, higher blood glucose level was observed in the infected group than in the noninfected group. A positive Δ value indicated a slow glucose tolerance status, which could lead to the conclusion that the infection caused slow and acceleration of diabetes in mice as observed in infection of prediabetic NOD mice, although in type 1 diabetes.^53^

Overall, for males on CHD, an improvement was observed in both infection conditions, also observed by Berbudi et al^54^ in diet‐induced obese mice. For males on HFD, a slight aggravation was observed in the infected group and an overall improvement in the noninfected group. Almost similar results were obtained by Miao et al^55^ in adult mice after HFD consumption. Interestingly, males of IL1912 on HFD show deterioration in their glucose tolerance ability in both infection conditions showing a positive ΔAUC_6–12_ value, which leads to the conclusion that this line is sensitive to HFD more than a bacterial infection. These results were consistent with earlier studies in mice by Winzell and Ahrén^56^ and Liu et al.^57^

These findings point to the linkage of the final BW of these lines in that the infected group reached a lower BW (g) value than the noninfected group on HFD among male mice, indicating that also in terms of BW these lines are resistant to infection combined with HFD. The results were corroborated by Strandberg et al^58^ and Wang et al.^59^

Diabetes mellitus is a chronic condition that develops over time and is preceded by the prediabetic state. How to exactly predict and diagnose this disease using ML is worth studying and is one of our objectives. The right diagnosis will lead us to introduce prevention strategies, and it must be noted that accurate prediction needs more indexes. The best result for the data set is above 0.80 in most CC lines, indicating ML can be used for prediction of diabetes, but finding suitable attributes, classifiers, and data mining methods is very important. Based on our results, we chose the method using all features, but still RF had the best result among the four classifiers, as also observed by Zou et al.^60^ Therefore, our observations provide valuable insight into the potential application of the AUC as a predictive measure for T2D and highlight the need. These ML methods have also been recently applied by Ben‐Assuli et al^61^ for faster diagnosis and treatment of NAFLD. Our results provide a significant resource for further studies to determine the causal relationship and the progression of T2D; therefore, the prospect of using personalized medicine is a promise. The results presented in this paper is the first step toward applying personalized/precision medicine approach based on early prediction and prevention approaches.

To our knowledge, this is the first study to examine the effect of the host genetic background on the development of a single disease or a combination of two or three diseases, including obesity and diabetes, using the CC mouse model. Finally, it is believed that assessing more CC lines and increasing the number of mice within each line as well as using both the multitrait and the multilocus analytical methods developed specifically for this genetically highly diverse reference population. This will enable future studies to map quantitative trait loci with unprecedented precision, allowing the direct identification of potential candidate genes and their multilocus epistatic interactions associated with susceptibility to obesity and T2D development.

## AUTHOR CONTRIBUTIONS

Iqbal M. Lone was involved in data analysis and writing the manuscript; Nadav Ben Nun was involved in statistical analysis; Aya Ghnaim was involved in data collection and analysis; Arne S. Schaefer was involved in project design and fund support; Yael Houri‐Haddad was involved in project design, execution, and fund support; Fuad A. Iraqi was involved in project design, data analysis, fund support, team supervision, and drafting and reviewing and approving the final version of the manuscript.

## FUNDING INFORMATION

Binational Science Foundation (BSF) grant number 2015077, German Israeli Science Foundation (GIF) grant I‐63‐410.20‐2017, Israeli Science Foundation (ISF) grant 1085/18, and core fund from Tel Aviv University.

## CONFLICT OF INTEREST STATEMENT

The authors declare no competing financial interests or other associations that may pose a conflict of interest (e.g., pharmaceutical stock ownership, consultancy). Fuad A. Iraq is an Editorial Board member of AMEM and a co‐author of this article. To minimize bias, he was excluded from all editorial decision‐making related to the acceptance of this article for publication.

## Supporting information


Appendix S1

## References

[1] Azevedo M , Alla S . Diabetes in sub‐saharan Africa: Kenya, Mali, Mozambique, Nigeria, South Africa and Zambia. Int J Diabetes Dev Ctries. 2008;28(4):101‐108. doi:10.4103/0973-3930.45268 20165596 PMC2822152

[2] Iancu I , Mota M , Iancu E . Method for the analysing of blood glucose dynamics in diabetes mellitus patients. IEEE International Conference on Automation, Quality and Testing, Robotics. Vol 3. IEEE; 2008:60‐65.

[3] Cox ME , Edelman D . Tests for screening and diagnosis of type 2 diabetes. Clin Diabetes. 2009;27(4):132‐138.

[4] American Diabetes Association . Diagnosis and classification of *Diabetes mellitus* . Diabetes Care. 2012;35(Suppl 1):S64‐S71. doi:10.2337/dc12-s064 22187472 PMC3632174

[5] Wu Y , Ding Y , Tanaka Y , Zhang W . Risk factors contributing to type 2 diabetes and recent advances in the treatment and prevention. Int J Med Sci. 2014;11(11):1185‐1200. 25249787 10.7150/ijms.10001 PMC4166864

[6] Lone IM , Iraqi FA . Genetics of murine type 2 diabetes and comorbidities. Mamm Genome. 2022;1‐6:421‐436. 10.1007/s00335-022-09948-x 35113203

[7] Twito O , Frankel M , Nabriski D . Impact of glucose level on morbidity and mortality in elderly with diabetes and pre‐diabetes. World J Diabetes. 2015;6(2):345‐351. 25789117 10.4239/wjd.v6.i2.345 PMC4360429

[8] De Candia P , Prattichizzo F , Garavelli S , et al. Type 2 diabetes: how much of an autoimmune disease? Front Endocrinol. 2019;10:451. 10.3389/fendo.2019.00451 PMC6620611 31333589

[9] WHO‐world health organization . Accessed January, 2020. https://www.who.int/health‐topics/diabetes

[10] Mathers CD , Loncar D . Projections of global mortality and bur‐ den of disease from 2002 to 2030. PLoS Med. 2006;3(11):442. 10.1371/journal.pmed.0030442 PMC1664601 17132052

[11] Reaven G . Role of insulin resistance in human disease. Diabetes. 1988;37:1595‐1607. doi:10.2337/diab.37.12.1595 3056758

[12] Buse JB , Ginsberg HN , Bakris GL , Clark NG , Costa F , Eckel R . Primary prevention of cardiovascular diseases in people with diabetes mellitus: a scientific statement from the American Heart Association and the American Diabetes Association. Circulation. 2007;115(1):114‐126. 17192512 10.1161/CIRCULATIONAHA.106.179294

[13] Chan JM , Rimm EB , Colditz GA , Stampfer MJ , Willett WC . Obesity, fat distribution, and weight gain as risk factors for clinical diabetes in men. Diabetes Care. 1994;17(9):961‐969. doi:10.2337/diacare.17.9.961 7988316

[14] Carey VJ , Walters EE , Colditz GA , et al. Body fat distribution and risk of non‐insulin‐dependent diabetes mellitus in women: the Nurses' health study. Am J Epidemiol. 1997;145(7):614‐619. 9098178 10.1093/oxfordjournals.aje.a009158

[15] Wang Y , Rimm EB , Stampfer MJ , Willett WC , Hu FB . Comparison of abdominal adiposity and overall obesity in predicting risk of type 2 diabetes among men. Am J Clin Nutr. 2005;81(3):555‐563. 15755822 10.1093/ajcn/81.3.555

[16] Karjalainen KM , Knuuttila ML , von Dickhoff KJ . Association of the severity of periodontal disease with organ complications in type 1 diabetic patients. J Periodontol. 1994;65(11):1067‐1072. 7853131 10.1902/jop.1994.65.11.1067

[17] Moore PA , Weyant RJ , Mongelluzzo MB , et al. Type 1 diabetes mellitus and oral health: assessment of periodontal disease. J Periodontol. 1999;70(4):409‐417. 10328653 10.1902/jop.1999.70.4.409

[18] Al‐Maskari AY , Al‐Maskari MY , Al‐Sudairy S . Oral manifestations and complications of diabetes mellitus: a review. Sultan Qaboos Univ Med J. 2011;11(2):179‐186. 21969888 PMC3121021

[19] Wen L , Duffy A . Factors influencing the gut microbiota, inflammation, and type 2 diabetes. J Nutr. 2017;147(7):1468S‐1475S. 28615382 10.3945/jn.116.240754 PMC5483960

[20] Peng CH , Yang YS , Chan KC , Kornelius E , Chiou JY , Huang CN . Periodontal treatment and the risks of cardiovascular disease in patients with type 2 diabetes: a retrospective cohort study. Intern Med. 2017;56(9):1015‐1021. 28458305 10.2169/internalmedicine.56.7322 PMC5478560

[21] Lone IM , Zohud O , Nashef A , et al. Dissecting the complexity of skeletal‐malocclusion‐associated phenotypes: mouse for the rescue. Int J Mol Sci. 2023;24(3):2570. doi:10.3390/ijms24032570 36768894 PMC9916875

[22] Atamni HJ , Mahmoud E , Yaser S , Aysar N , Iraqi FA . The collaborative cross mouse genetic reference population designed for dissecting complex traits. Chin J Comp Med. 2016;26:1‐19.

[23] Atamni HJ , Mott R , Soller M , Iraqi FA . High‐fat‐diet induced development of increased fasting glucose levels and impaired response to intraperitoneal glucose challenge in the collaborative cross mouse genetic reference population. BMC Genet. 2016c;17:10. doi:10.1186/s12863-015-0321-x 26728312 PMC4700737

[24] Yehia R , Lone IM , Yehia I , Iraqi FA . Studying the Pharmagenomic effect of Portulaca oleracea extract on anti‐diabetic therapy using the collaborative cross mice. Phytomedicine Plus. 2023;3(1):100394.

[25] Lee BJ , Kim JY . Identification of type 2 diabetes risk factors using phenotypes consisting of anthropometry and triglycerides based on machine learning. IEEE J Biomed Health Inform. 2015;20(1):39‐46. doi:10.1109/JBHI.2015.2396520 25675467

[26] Alghamdi M , Al‐Mallah M , Keteyian S , Brawner C , Ehrman J , Sakr S . Predicting diabetes mellitus using SMOTE and ensemble machine learning approach: the Henry ford ExercIse testing (FIT) project. PLoS ONE. 2017;12(7):e0179805. doi:10.1371/journal.pone.0179805 28738059 PMC5524285

[27] Kavakiotis I , Tsave O , Salifoglou A , Maglaveras N , Vlahavas I , Chouvarda I . Machine learning and data mining methods in diabetes research. Comput Struct Biotechnol J. 2017;15:104‐116. doi:10.1016/j.csbj.2016.12.005 28138367 PMC5257026

[28] Razavian N , Blecker S , Schmidt AM , Smith‐McLallen A , Nigam S , Sontag D . Population‐level prediction of type 2 diabetes from claims data and analysis of risk factors. Big Data. 2015;3(4):277‐287. doi:10.1089/big.2015.0020 27441408

[29] Iraqi FA , Churchill G , Mott R . The collaborative cross, developing a resource for mammalian systems genetics: a status report of the Wellcome Trust cohort. Mamm Genome. 2008;19(6):379‐381. 18521666 10.1007/s00335-008-9113-1

[30] Iraqi FA , Athamni H , Dorman A , et al. Heritability and coefficient of genetic variation analyses of phenotypic traits provide strong basis for high‐resolution QTL mapping in the collaborative cross mouse genetic reference population. Mamm Genome. 2014;25(3):109‐119. 24445421 10.1007/s00335-014-9503-5

[31] Garcia‐Gonzalez F , Simmons LW , Tomkins JL , Kotiaho JS , Evans JP . Comparing evolvabilities: common errors surrounding the calculation and use of coefficients of additive genetic variation. Evol Int J Org Evol. 2012;66(8):2341‐2349. 10.1111/j.1558-5646.2011.01565.x 22834736

[32] Houle D . Comparing evolvability and variability of quantitative traits. Genetics. 1992;130(1):195‐204. 1732160 10.1093/genetics/130.1.195 PMC1204793

[33] Salzberg SL . Machine Learning by J. Ross Quinlan. Morgan Kaufmann Publishers, Inc. 1993; 1994;1:6.

[34] Breiman L . Random forests. Machine Learning. 2001;45:5‐32. doi:10.1023/A:1010933404324

[35] Lin C , Chen W , Qiu C , Wu Y , Krishnan S , Zou Q . LibD3C: ensemble classifiers with a clustering and dynamic selection strategy. Neurocomputing. 2014;123:424‐435. doi:10.1016/j.neucom.2013.08.004

[36] Svetnik V , Liaw A , Tong C , Culberson JC , Sheridan RP , Feuston BP . Random forest: a classification and regression tool for compound classification and QSAR modeling. J Chem Inf Comput Sci. 2003;43(6):1947‐1958. doi:10.1021/ci034160g 14632445

[37] Zhao X , Zou Q , Liu B , Liu X . Exploratory predicting protein folding model with random forest and hybrid features. Curr Proteom. 2014;11(4):289‐299. doi:10.2174/157016461104150121115154

[38] Liao Z , Ju Y , Zou Q . Prediction of G protein‐coupled receptors with SVM‐prot features and random forest. Scientifica. 2016;2016:8309253. doi:10.1155/2016/8309253 27529053 PMC4978840

[39] Kohavi R . A study of cross‐validation and bootstrap for accuracy estimation and model selection. IJCAI. 1995;14(2):1137‐1145.

[40] Refaeilzadeh P , Tang L , Liu H . Cross‐validation. In: Liu L , Özsu MT , eds. Encyclopedia of Database Systems. Springer; 2016:532‐538.

[41] Su ZD , Huang Y , Zhang ZY , et al. iLoc‐lncRNA: predict the subcellular location of lncRNAs by incorporating octamer composition into general PseKNC. Bioinformatics. 2018;34(24):4196‐4204. doi:10.1093/bioinformatics/bty508. [Epub ahead of print]. 29931187

[42] Tang H , Zhao YW , Zou P , et al. HBPred: a tool to identify growth hormone‐binding proteins. Int J Biol Sci. 2018;14(8):957‐964. doi:10.7150/ijbs.24174 29989085 PMC6036759

[43] Kim JH . Estimating classification error rate: repeated cross‐validation, repeated hold‐out and bootstrap. Comput Stat Data Anal. 2009;53(11):3735‐3745. doi:10.1016/j.csda.2009.04.009

[44] Jagannathachary S , Kamaraj D . Obesity and periodontal disease. J Indian Soc Periodontol. 2010;14(2):96‐100. 21691545 10.4103/0972-124X.70827 PMC3110475

[45] Leitner DR , Frühbeck G , Yumuk V , et al. Obesity and type 2 diabetes: two diseases with a need for combined treatment strategies‐EASO can lead the way. Obes Facts. 2017;10(5):483‐492. doi:10.1159/000480525 29020674 PMC5741209

[46] Yang W , Lu J , Weng J , et al. Prevalence of diabetes among men and women in China. N Engl J Med. 2010;362(12):1090‐1101. 20335585 10.1056/NEJMoa0908292

[47] Shen X , Vaidya A , Wu S , Gao X . The diabetes epidemic in China: an integrated review of national surveys. Endocr Pract. 2016;22(9):1119‐1129. doi:10.4158/EP161199.RA 27295015 PMC5567749

[48] NCD Risk Factor Collaboration (NCD‐RisC). Africa Working Group . Trends in obesity and diabetes across Africa from 1980 to 2014: an analysis of pooled population‐based studies. Int J Epidemiol. 2017;46(5):1421‐1432. 28582528 10.1093/ije/dyx078 PMC5837192

[49] Kwok AJ , Mentzer A , Knight JC . Host genetics and infectious disease: new tools, insights and translational opportunities. Nat Rev Genet. 2021;22(3):137‐153. doi:10.1038/s41576-020-00297-6 33277640 PMC7716795

[50] Karkar L , Abu‐Toamih Atamni HJ , Milhem A , Houri‐Haddad Y , Iraqi FA . Assessing the host genetic background effects on type 2 diabetes and obesity development in response to mixed–oral bacteria and high‐fat diet using the collaborative cross mouse model. Animal Model Exp Med. 2020;3(2):152‐159. doi:10.1002/ame2.12117 32613174 PMC7323698

[51] Cignarella A , Bolego C . Mechanisms of estrogen protection in diabetes and metabolic disease. Horm Mol Biol Clin Investig. 2010;4(2):575‐580. doi:10.1515/HMBCI.2010.084 25961234

[52] Milhem A , Abu Toamih‐Atamni HJ , Karkar L , Houri‐Haddad Y , Iraqi FA . Studying host genetic background effects on multimorbidity of intestinal cancer development, type 2 diabetes and obesity in response to oral bacterial infection and high‐fat diet using the collaborative cross (CC) lines. Animal Model Exp Med. 2021;4(1):27‐39. 33738434 10.1002/ame2.12151 PMC7954829

[53] Christen U , Bender C , von Herrath MG . Infection as a cause of type 1 diabetes? Curr Opin Rheumatol. 2012;24(4):417‐423. doi:10.1097/BOR.0b013e3283533719. PMID: 22504578; PMCID: PMC4828240. 22504578 PMC4828240

[54] Berbudi A , Surendar J , Ajendra J , et al. Filarial infection or antigen administration improves glucose tolerance in diet‐induced obese mice. J Innate Immun. 2016;8(6):601‐616. doi:10.1159/000448401 27544668 PMC6743339

[55] Miao ZH , Zhou WX , Cheng RY , et al. Dysbiosis of intestinal microbiota in early life aggravates high‐fat diet induced dysmetabolism in adult mice. BMC Microbiol. 2021;21(1):1‐2. doi:10.1186/s12866-021-02263-6 34238228 PMC8268513

[56] Winzell MS , Ahrén B . The high‐fat diet–fed mouse: a model for studying mechanisms and treatment of impaired glucose tolerance and type 2 diabetes. Diabetes. 2004;53(suppl_3):S215‐S219. 15561913 10.2337/diabetes.53.suppl_3.s215

[57] Liu Y , Yang K , Jia Y , et al. Gut microbiome alterations in high‐fat‐diet‐fed mice are associated with antibiotic tolerance. Nat Microbiol. 2021;6(7):874‐884. doi:10.1038/s41564-021-00912-0 34017107

[58] Strandberg L , Verdrengh M , Enge M , et al. Mice chronically fed high‐fat diet have increased mortality and disturbed immune response in sepsis. PloS ONE. 2009;4(10):e7605. doi:10.1371/journal.pone.0007605 19865485 PMC2765728

[59] Wang R , Chen D , Wang F , et al. An insight into the exploration of proliferation of antibiotic resistance genes in high‐fat diet induced obesity mice. Genomics. 2021;113(4):2503‐2512. 34089783 10.1016/j.ygeno.2021.05.041

[60] Zou Q , Qu K , Luo Y , Yin D , Ju Y , Tang H . Predicting diabetes mellitus with machine learning techniques. Front Genet. 2018;9:515. doi:10.3389/fgene.2018.00515 30459809 PMC6232260

[61] Ben‐Assuli O , Jacobi A , Goldman O , et al. Stratifying individuals into non‐alcoholic fatty liver disease risk levels using time series machine learning models. J Biomed Inform. 2022;126:1‐6. doi:10.1016/j.jbi.2022.103986 35007752

