# Supplementary material for: High‐fat diet and oral infection induced type 2 diabetes and obesity development under different genetic backgrounds
Source: Animal Model Exp Med. 2023 Apr 7;6(2):131–45. doi: 10.1002/ame2.12311 (PMC10158944; doi:10.1002/ame2.12311)
Supplement: Supplementary file 1 — Appendix S1 [file AME2-6-131-s001.docx]

# Dynamics of body weight (BW) changes during the experimental period.


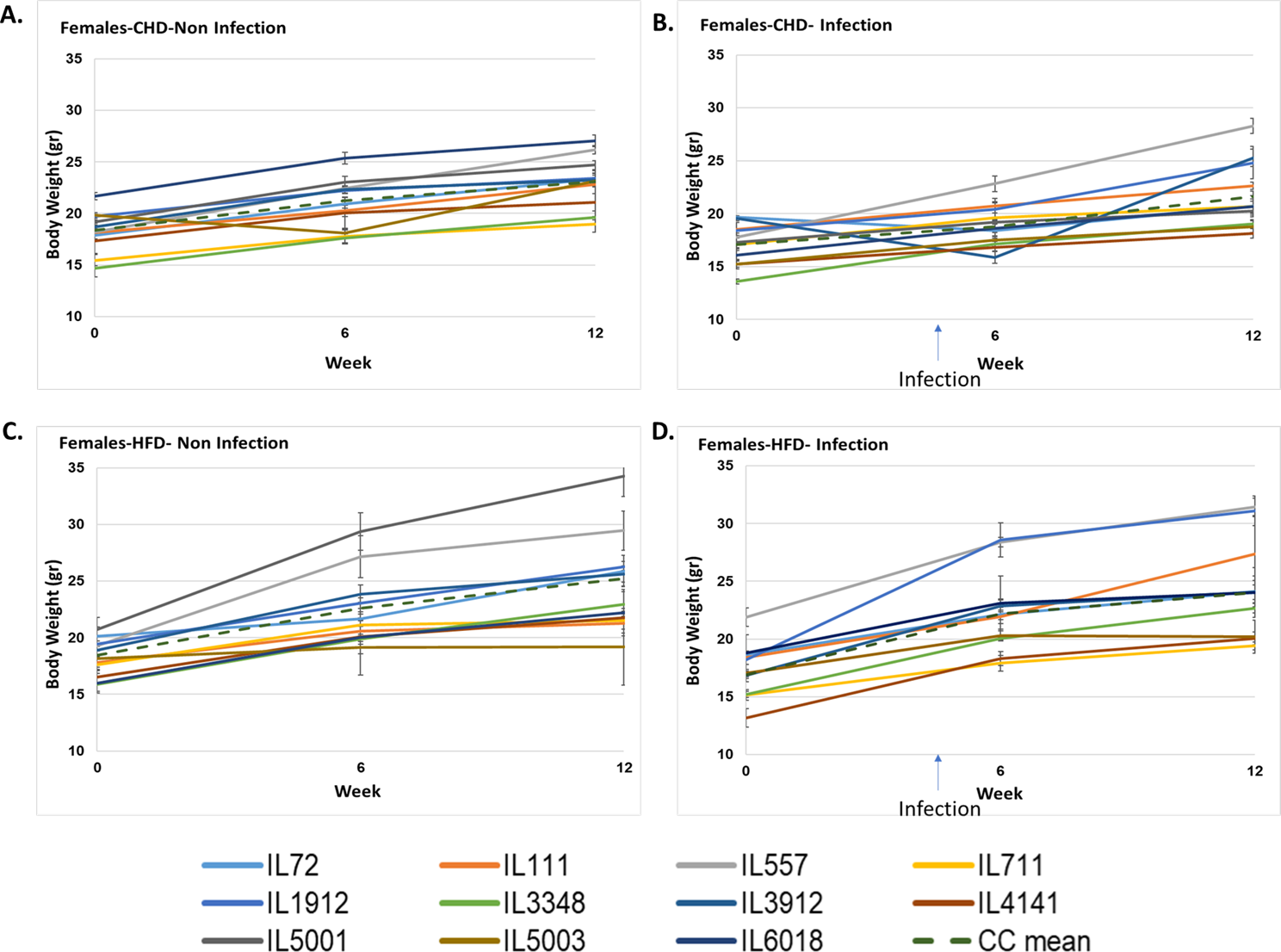


**Figure S1: Dynamics of BW changes in grams (gr) for females during the 12 weeks of the experiment in both diet groups and infection condition.**

Figures S1A and S1B show changes of BW in grams (gr) in the CHD group. Figure S1A shows the non-infection condition and figure 4B shows the infection condition. Figures S1C and S1D show changes of BW in the HFD group. Figure S1C shows the non-infection condition and figure S1D shows the infection condition. The *X-axis* represents weeks on the dietary challenge/infection; the *Y-axis* represents BW (gr).


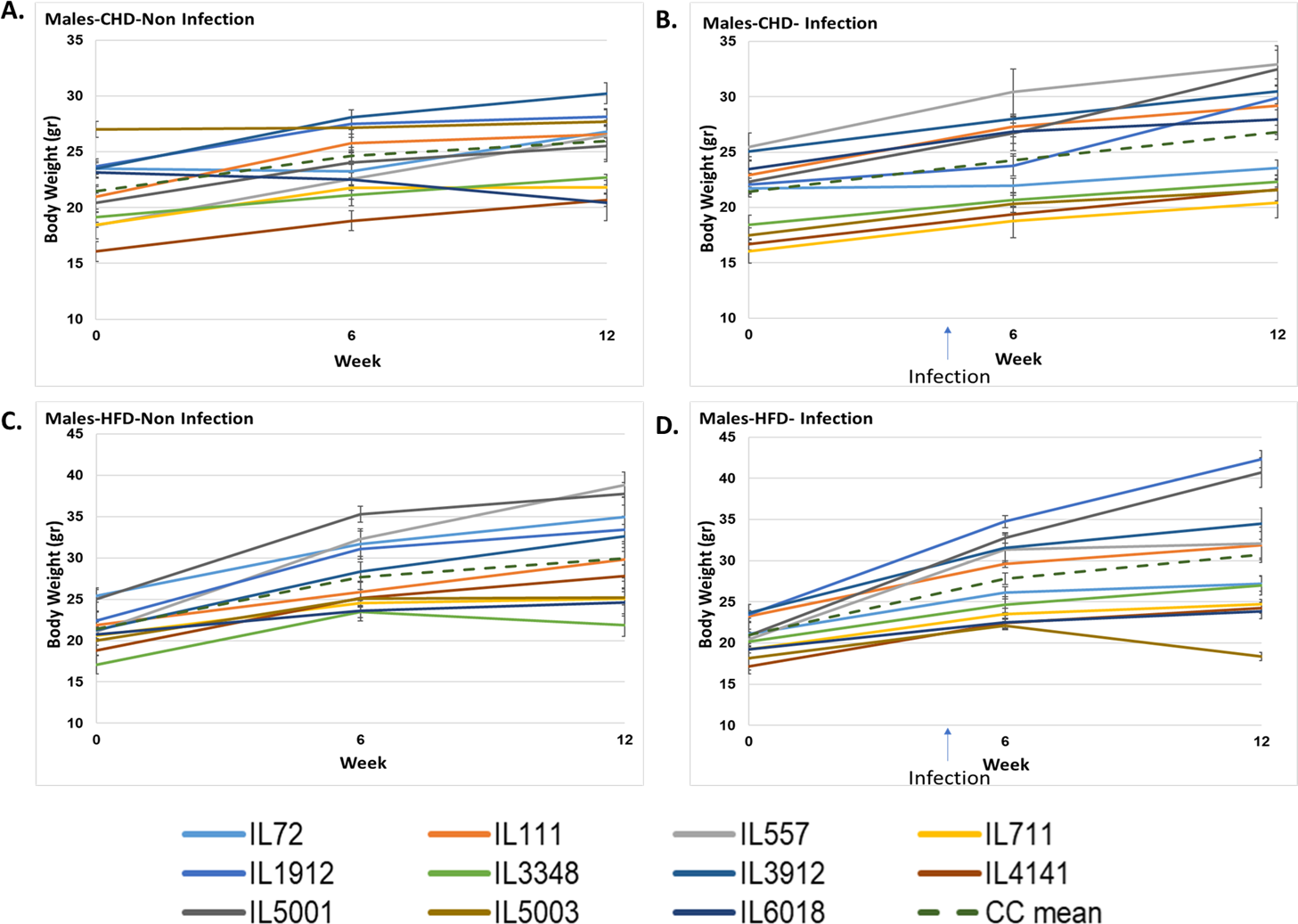


# Figure S2: Dynamics of BW changes in grams (gr) for males during the 12 weeks of the experiment in both diet groups and each infection condition.

Figures S2A and S2B show changes of BW in grams (gr) in the CHD group. Figure S2A shows the non-infection condition and Figure S2B show the infection condition. Figures S2C and S2D show changes in BW in the HFD group. Figure 5C shows the non-infection condition and Figure S2D shows the Infection condition. The *X-axis* represents weeks on the dietary challenge/infection; the *Y-axis* represents BW (gr).

# Percentage body weight changes.


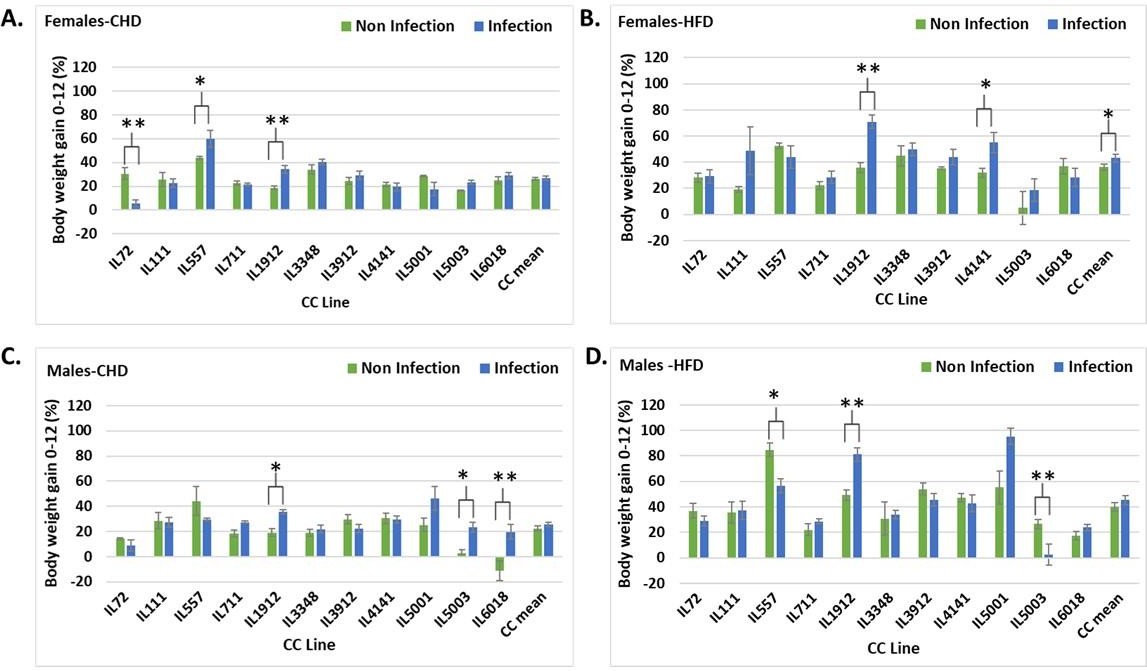

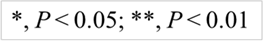


**Figure S3:** Percentage BW changes in grams (gr) between week 0-12 of 11 CC lines separately for females and males, on CHD and on HFD while infected and non-infected status as presented. Figures S3A and S3B show percentage BW changes between weeks 0-12 (±SE) for CHD and HFD of females respectively, by lines. Figures S3C and S3D show percentage BW changes between week 0-12 (±SE) for CHD and HFD of males respectively, by lines. The *X- axis* represents the different CC lines; the *Y-axis* represents the percentage of BW changes between weeks 0-12.

# Glucose tolerance ability changes (mg/dL* min) in both dietary challenge groups and infection conditions.


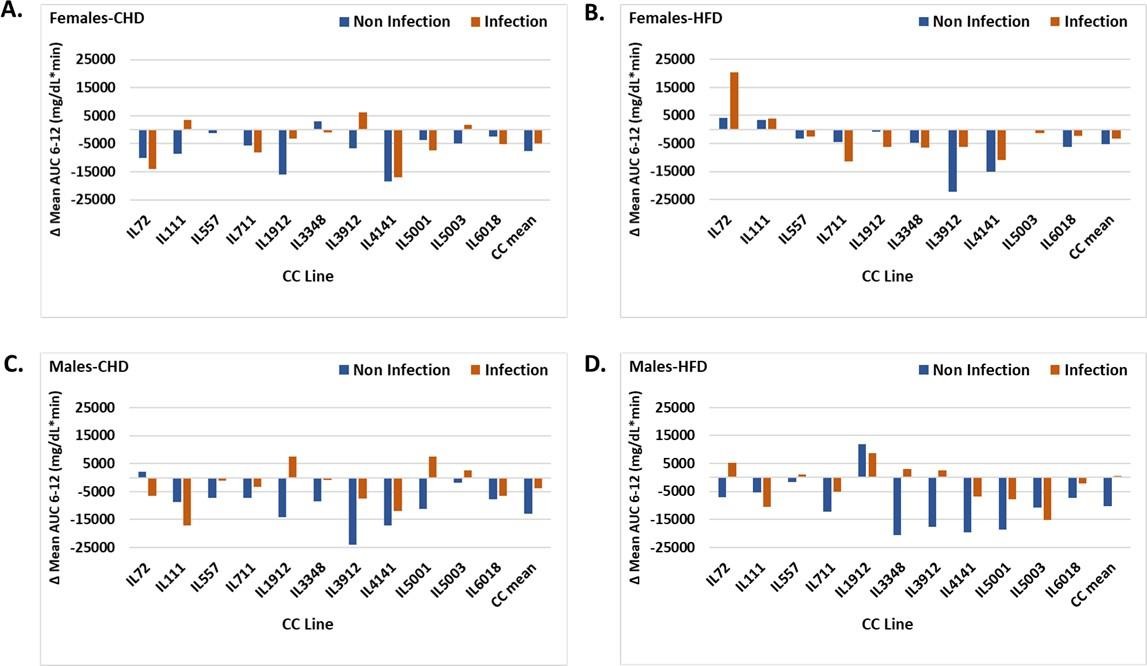

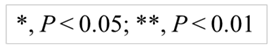


Negative Δ value = improvement Positive Δ value = deterioration

# Δ Mean AUC6-12= Mean AUC12- Mean AUC6 Per each Line

**Figure S4:** Variations in glucose tolerance ability in response to dietary and infection challenges of female and male mice of 11 different CC lines. Figures S4A and S4B present dynamics of AUC changes of female’s mice of the different 11 CC lines maintained on CHD and HFD regimes, respectively, while infected and non-infected status as presented. Figures S4C and S4D present dynamics of AUC changes of male’s mice of the different 11 CC lines maintained on the CHD and HFD, respectively, while infected and non-infected status as presented. The X*-axis* represents the different CC lines; the Y-*axis* represents the delta mean AUC (min*mg/dL).

# Fasting Blood Glucose changes.


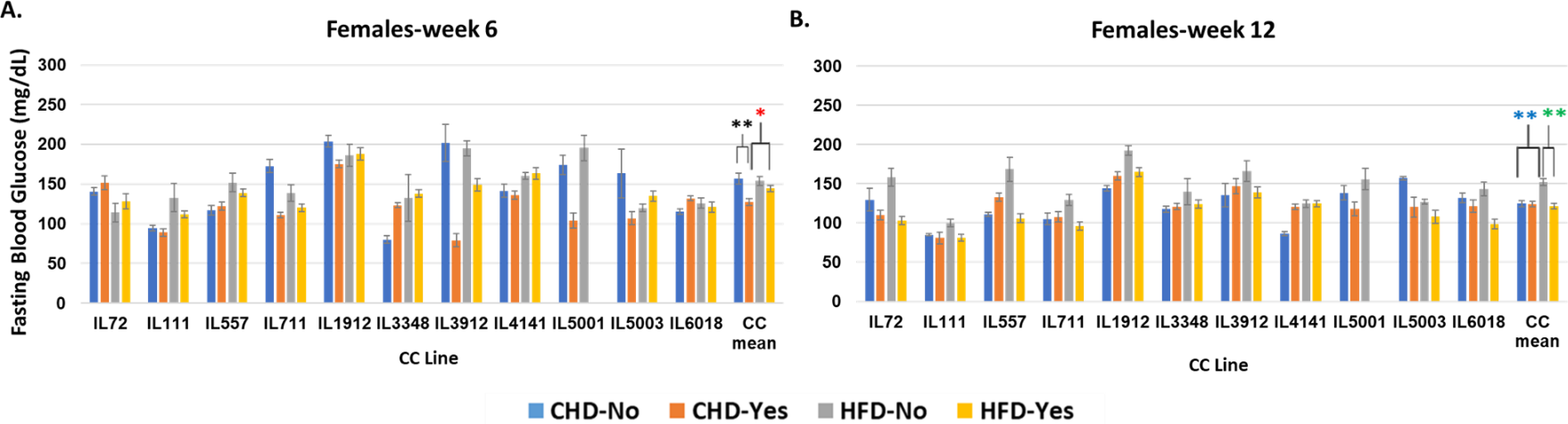


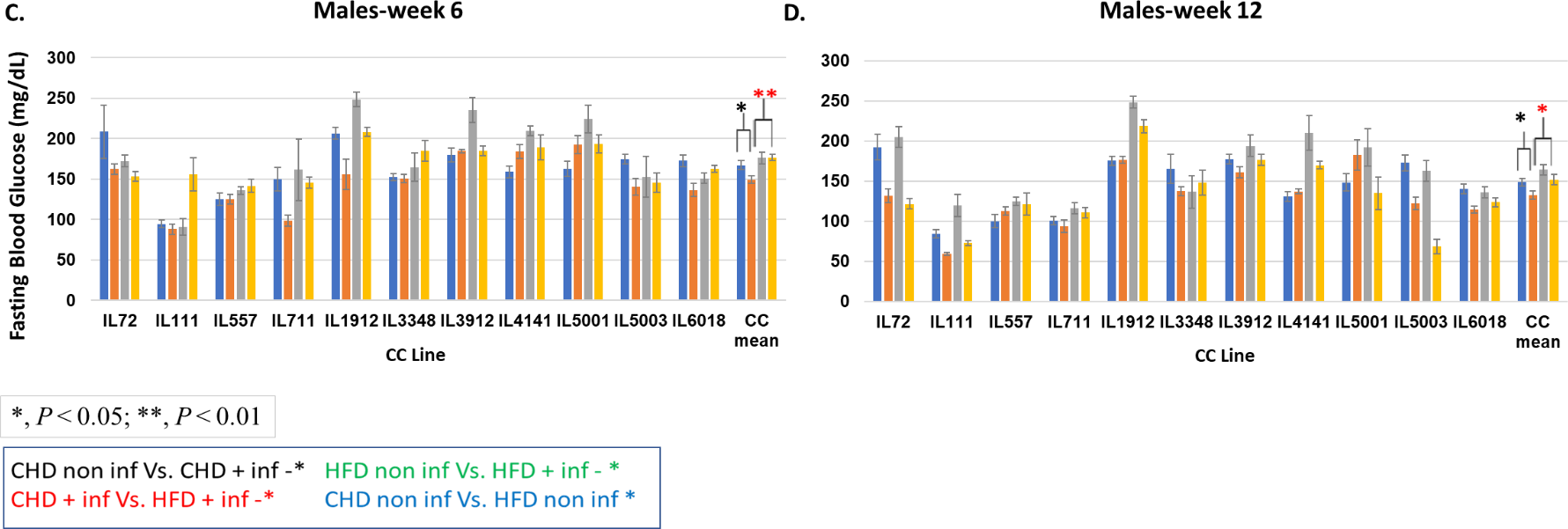


**Figure S5**: Fasting blood glucose (FBG) (mg/dL) levels at week 6 and at the end time of the experiment (week 12) on the dietary and Infection challenges as presented.

FBG (mg/dL) of female (Figures S5A and S5B) and male (Figures S5C and S5D) mice of 11 different CC lines, after 6 and 12 weeks among the four experimental groups, measured at time 0 before IPGTT glucose injection. The X*-axis* represents the different CC lines; the Y-*axis* represents fasting blood glucose (mg/dL).

The heatmaps of all the individual CC lines presented in this supplementary group file 2 (SG2).

**Heatmaps by Sex, Diet and Infection**

Female – CHD – No inf


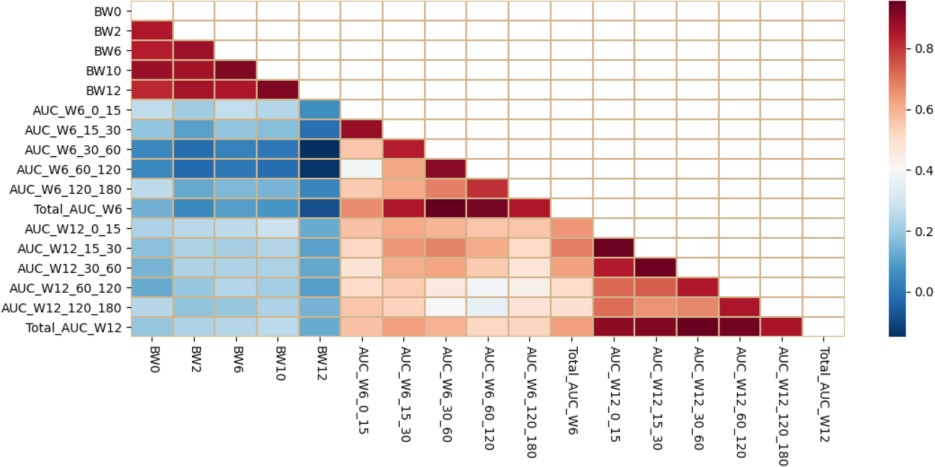


**Figure S6:** Heat map showing correlations between sex, diet and infection: BW and glucose tolerance referred here as AUC, at week 6 and week 12 of the experiment for females among the different conditions of the experiment. Figure presents the non-infection condition, and each map presents the dietary challenges, CHD. According to the color key, the correlation coefficient between -1≤ r ≤1 is significant at p<0.01 (**) and p<0.05 (*).

Female – CHD – Inf


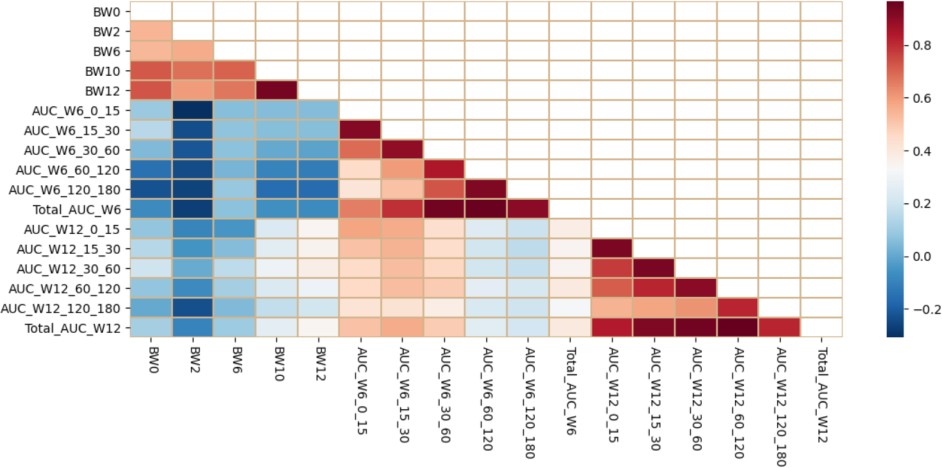


**Figure S7:** Heat map showing correlations between sex, diet and infection: BW and glucose tolerance referred here as AUC, at week 6 and week 12 of the experiment for females among the different conditions of the experiment. Figure presents the infection condition, and each map presents the dietary challenges, CHD. According to the color key, the correlation coefficient between -1≤ r ≤1 is significant at p<0.01 (**) and p<0.05 (*).

Female – HFD – No inf


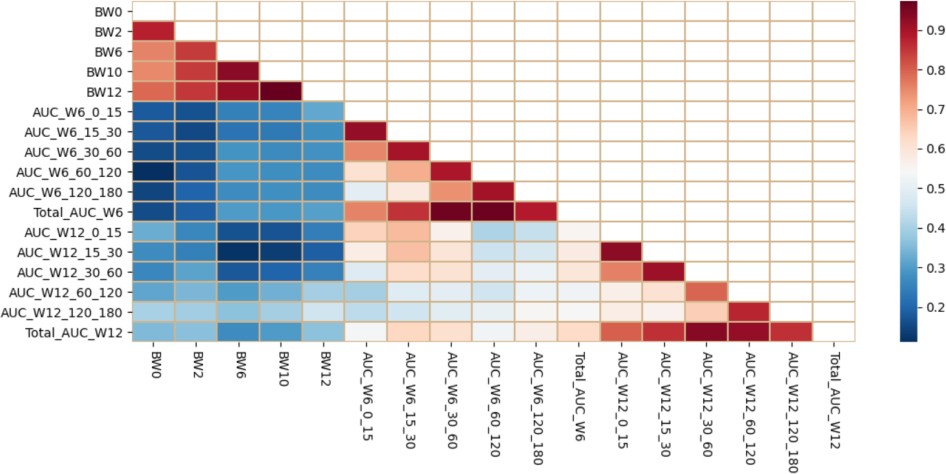


**Figure S8:** Heat map showing correlations between sex, diet and infection: BW and glucose tolerance referred here as AUC, at week 6 and week 12 of the experiment for females among the different conditions of the experiment. Figure presents the non-infection condition, and each map presents the dietary challenges, HFD. According to the color key, the correlation coefficient between -1≤ r ≤1 is significant at p<0.01 (**) and p<0.05 (*).

Female – HFD – Inf


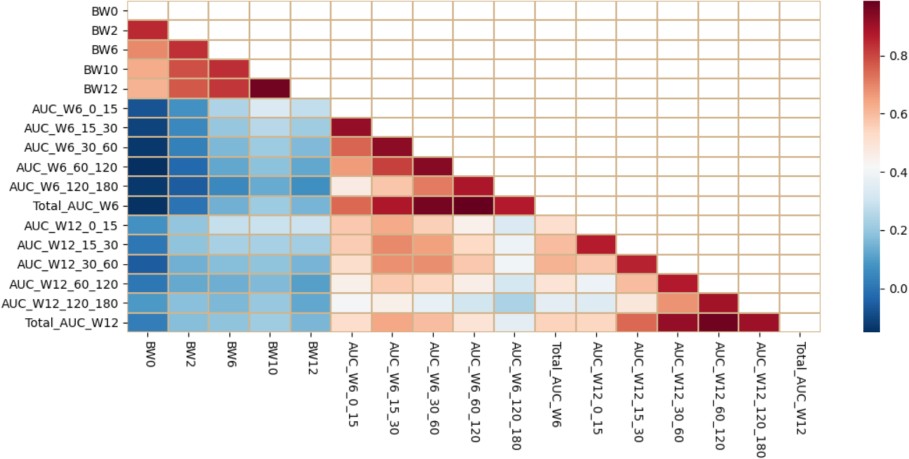


**Figure S9:** Heat map showing correlations between sex, diet and infection: BW and glucose tolerance referred here as AUC, at week 6 and week 12 of the experiment for females among the different conditions of the experiment. Figure presents the infection condition, and each map presents the dietary challenges, HFD. According to the color key, the correlation coefficient between -1≤ r ≤1 is significant at p<0.01 (**) and p<0.05 (*).

Male – CHD – No inf


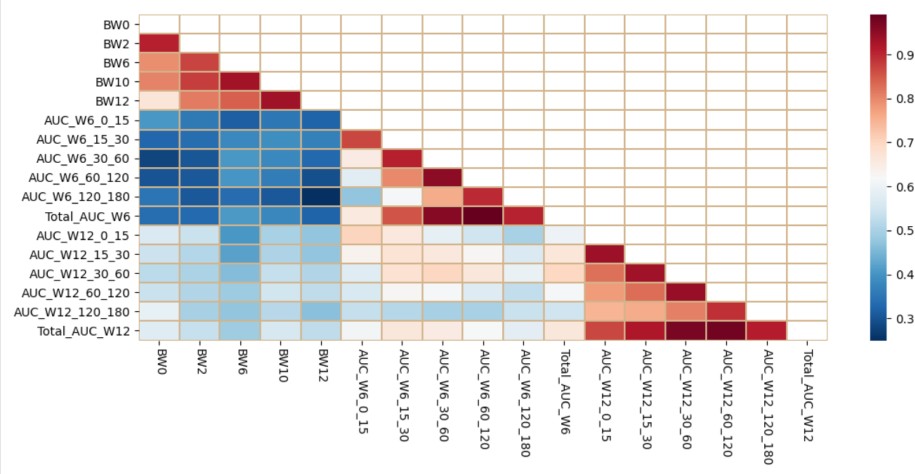


**Figure S10:** Heat map showing correlations between sex, diet and infection: BW and glucose tolerance referred here as AUC, at week 6 and week 12 of the experiment for males among the different conditions of the experiment. Figure presents the non-infection condition, and each map presents the dietary challenges, CHD. According to the color key, the correlation coefficient between -1≤ r ≤1 is significant at p<0.01 (**) and p<0.05 (*).

Male – CHD – Inf


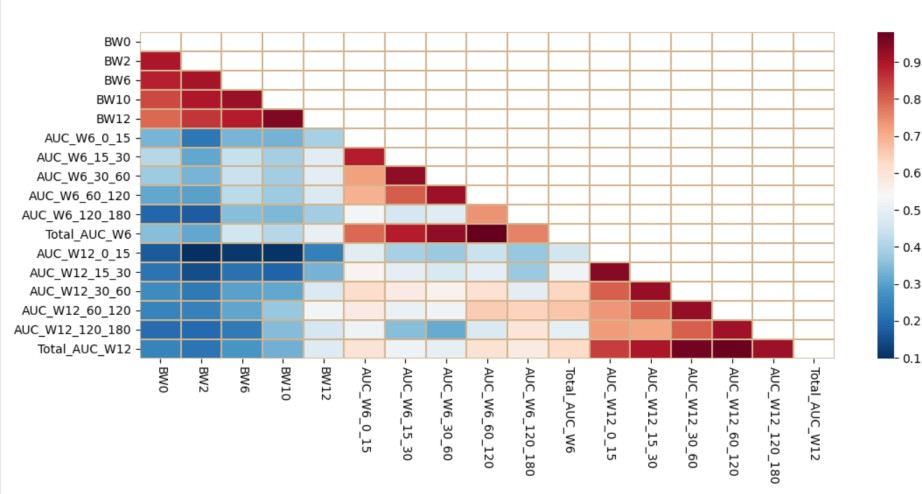


**Figure S11:** Heat map showing correlations between sex, diet and infection: BW and glucose tolerance referred here as AUC, at week 6 and week 12 of the experiment for males among the different conditions of the experiment. Figure presents the infection condition, and each map presents the dietary challenges, CHD. According to the color key, the correlation coefficient between -1≤ r ≤1 is significant at p<0.01 (**) and p<0.05 (*).

Male – HFD – No inf


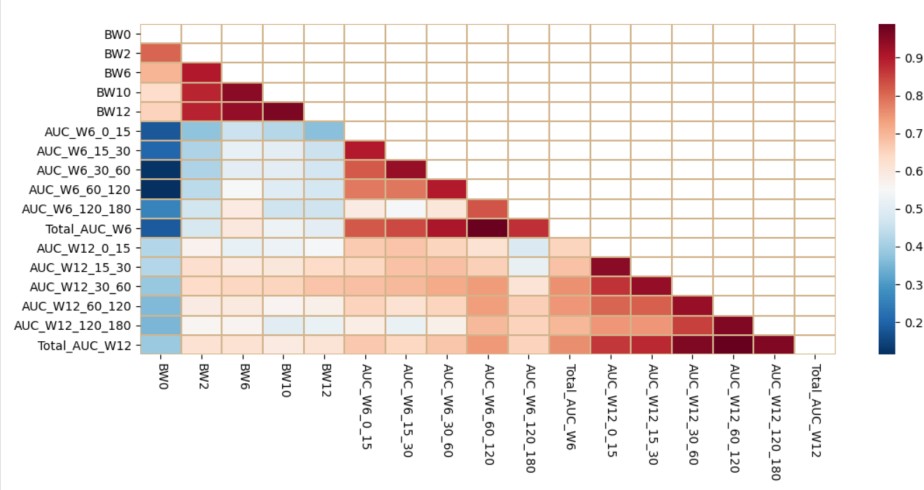


**Figure S12:** Heat map showing correlations between sex, diet and infection: BW and glucose tolerance referred here as AUC, at week 6 and week 12 of the experiment for males among the different conditions of the experiment. Figure presents the non-infection condition, and each map presents the dietary challenges, HFD. According to the color key, the correlation coefficient between -1≤ r ≤1 is significant at p<0.01 (**) and p<0.05 (*).

Male – HFD – Inf


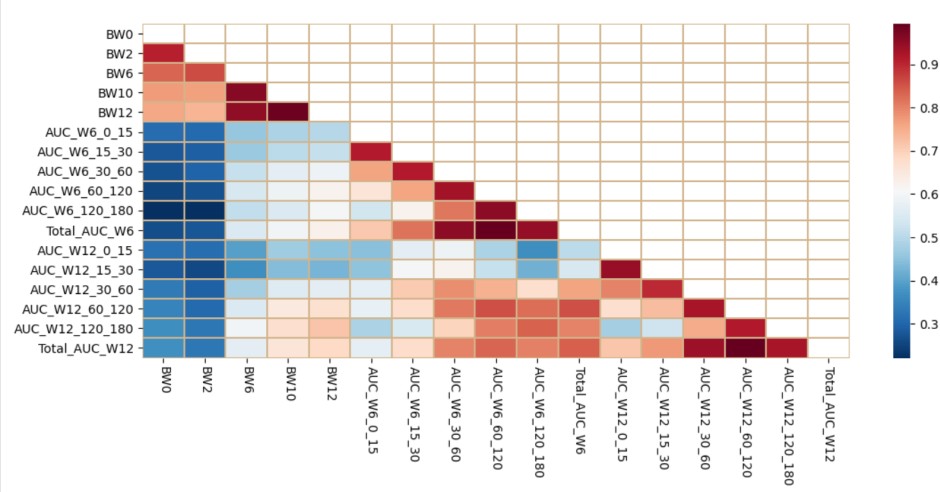


**Figure S13:** Heat map showing correlations between sex, diet and infection: BW and glucose tolerance referred here as AUC, at week 6 and week 12 of the experiment for males among the different conditions of the experiment. Figure presents the infection condition, and each map presents the dietary challenges, HFD. According to the color key, the correlation coefficient between -1≤ r ≤1 is significant at p<0.01 (**) and p<0.05 (*)

**Heatmaps By Lines and Sex**

Line 72 – F


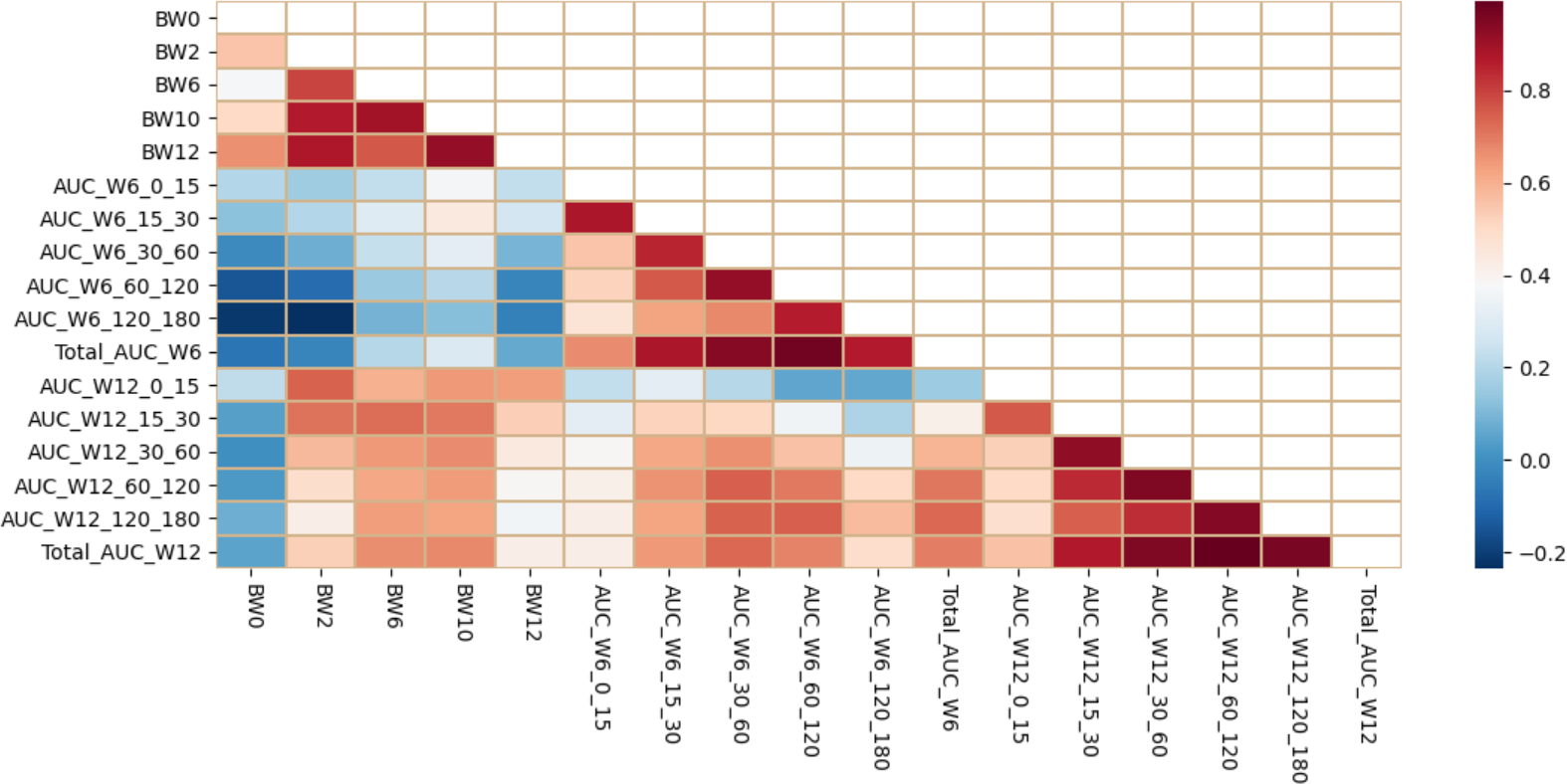


**Figure S14:** Heat map showing correlations between sex and individual CC line: BW and glucose tolerance referred here as AUC, at week 6 and week 12 of the experiment for females of line 72 among the different conditions of the experiment. Each map presents the influence of BW on AUC. According to the color key, the correlation coefficient between -1≤ r ≤1 is significant at p<0.01 (**) and p<0.05 (*).

Line 72 – M


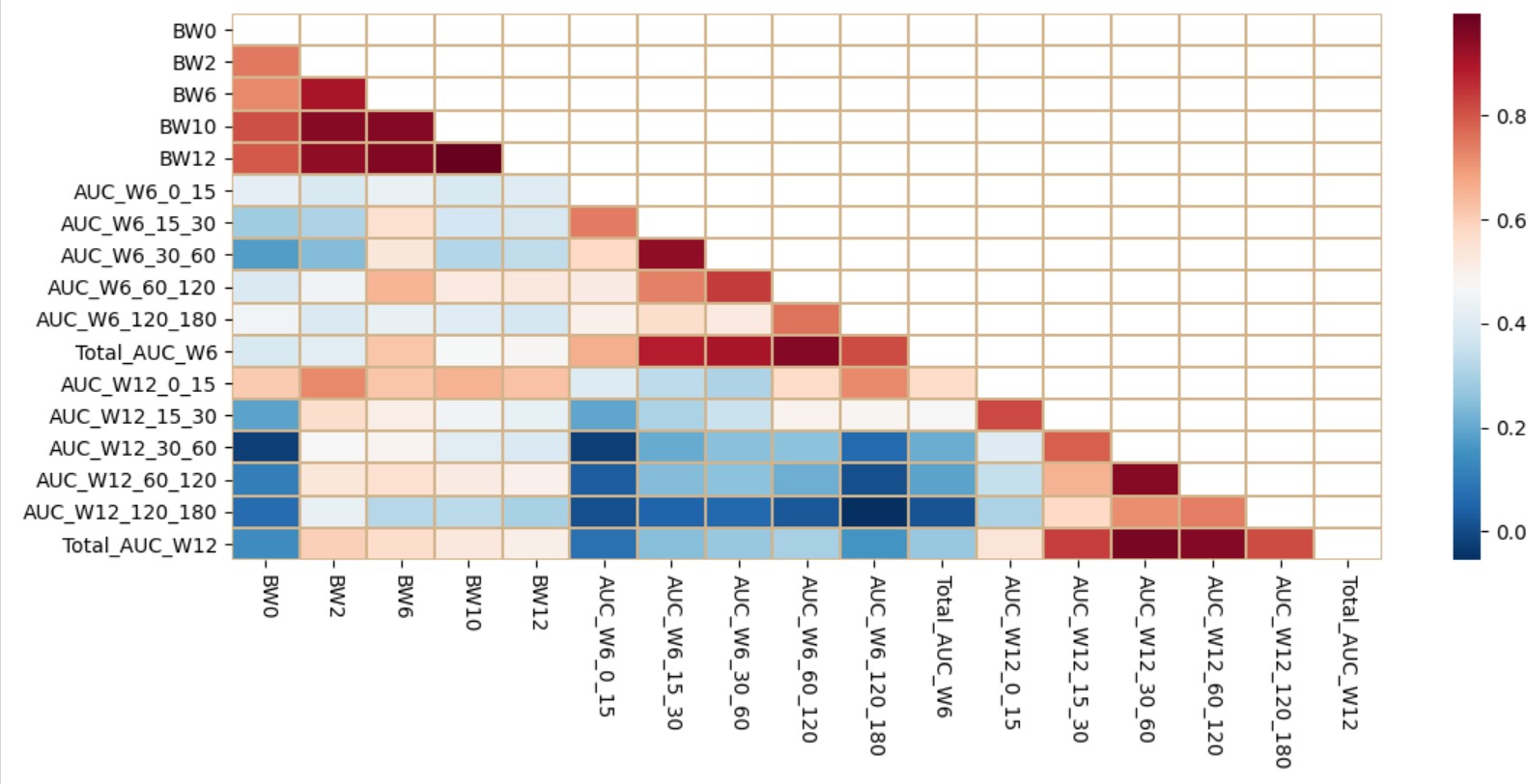


**Figure S15:** Heat map showing correlations between sex and individual CC line: BW and glucose tolerance referred here as AUC, at week 6 and week 12 of the experiment for males of line 72 among the different conditions of the experiment. Each map presents the influence of BW on AUC. According to the color key, the correlation coefficient between -1≤ r ≤1 is significant at p<0.01 (**) and p<0.05 (*).

Line 711 – F


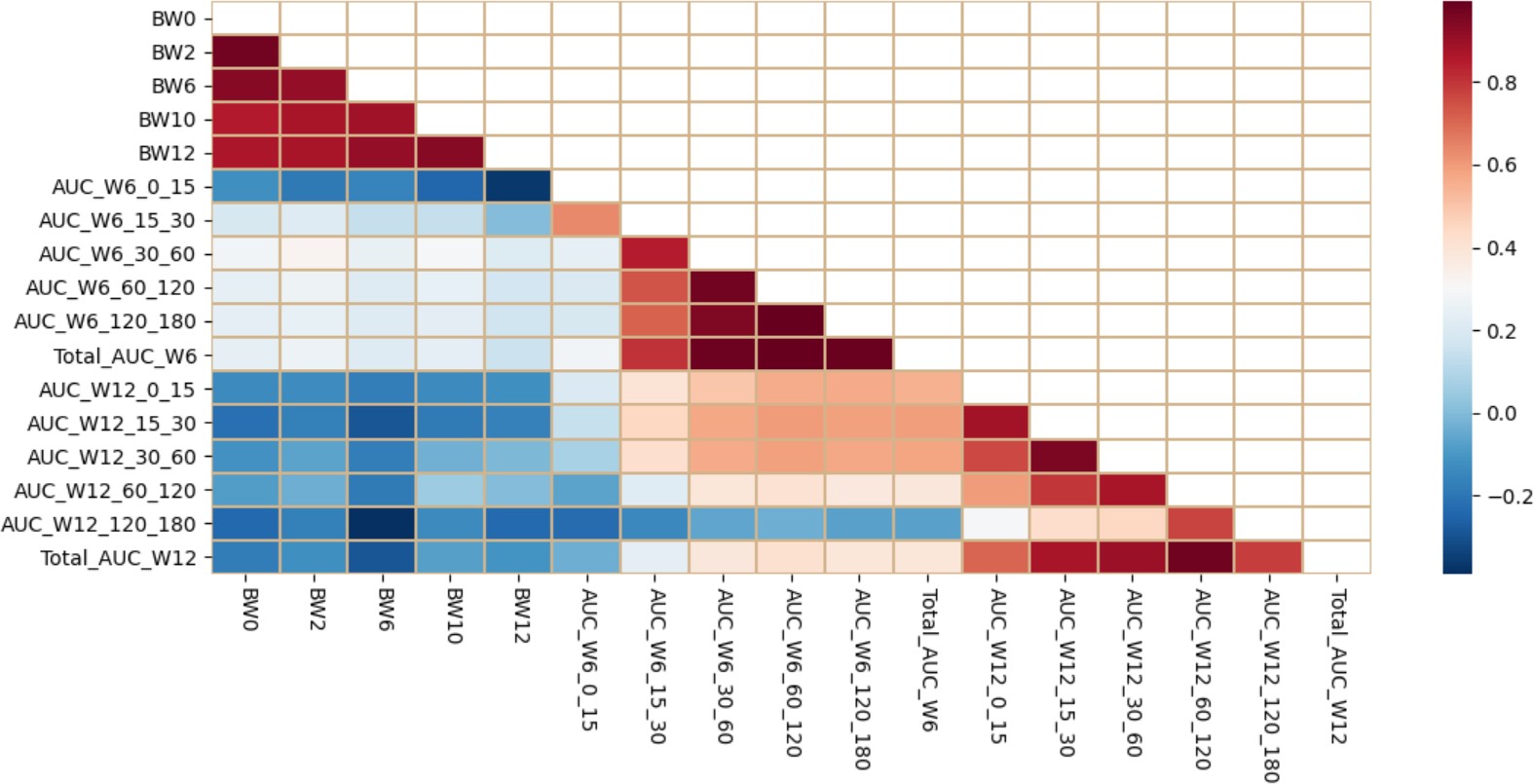


**Figure S16:** Heat map showing correlations between sex and individual CC line: BW and glucose tolerance referred here as AUC, at week 6 and week 12 of the experiment for females of line 711 among the different conditions of the experiment. Each map presents the influence of BW on AUC. According to the color key, the correlation coefficient between -1≤ r ≤1 is significant at p<0.01 (**) and p<0.05 (*).

Line 711 – M


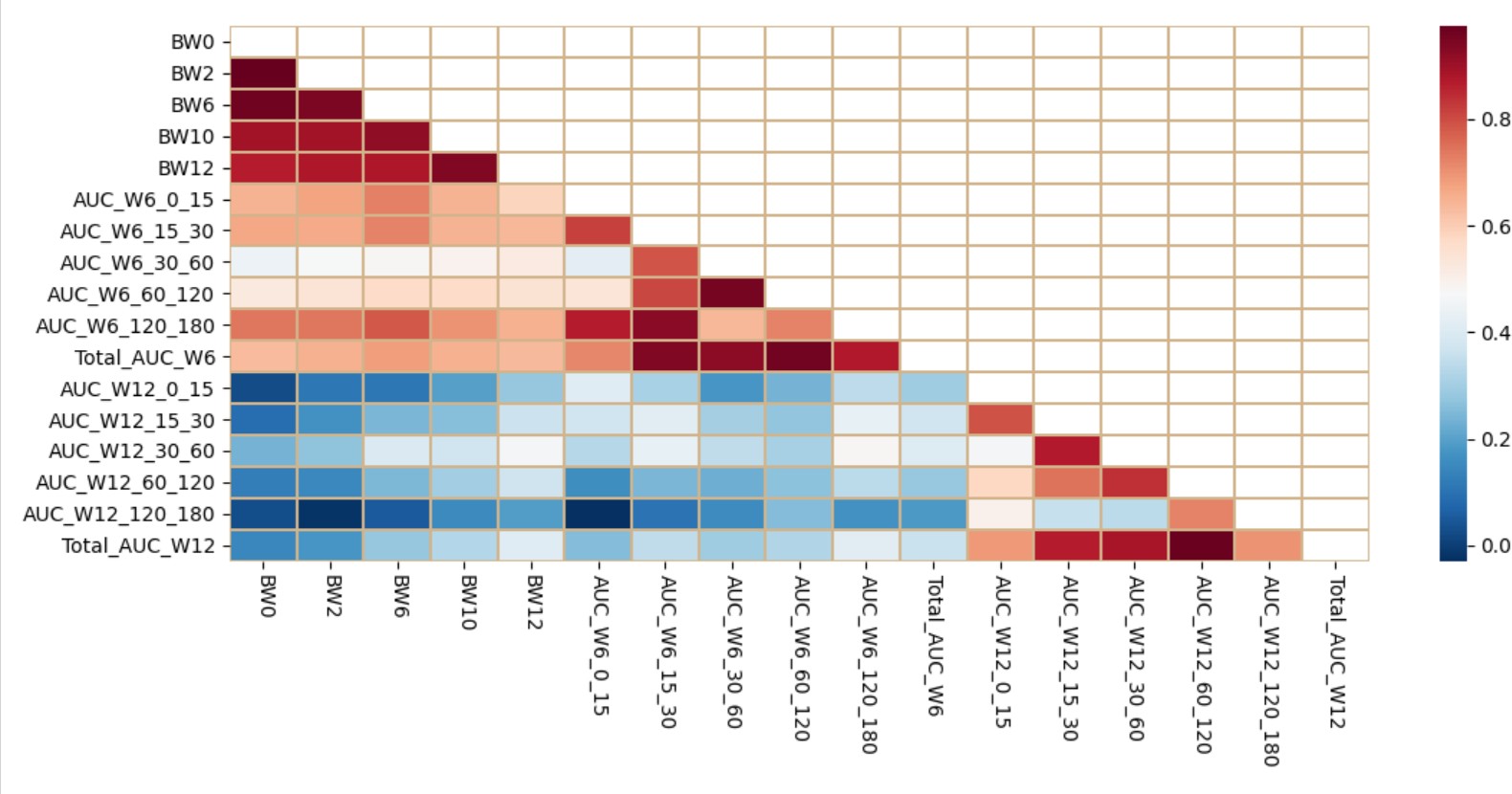


**Figure S17:** Heat map showing correlations between sex and individual CC line: BW and glucose tolerance referred here as AUC, at week 6 and week 12 of the experiment for males of line 711 among the different conditions of the experiment. Each map presents the influence of BW on AUC. According to the color key, the correlation coefficient between -1≤ r ≤1 is significant at p<0.01 (**) and p<0.05 (*).

Line 1912 – F


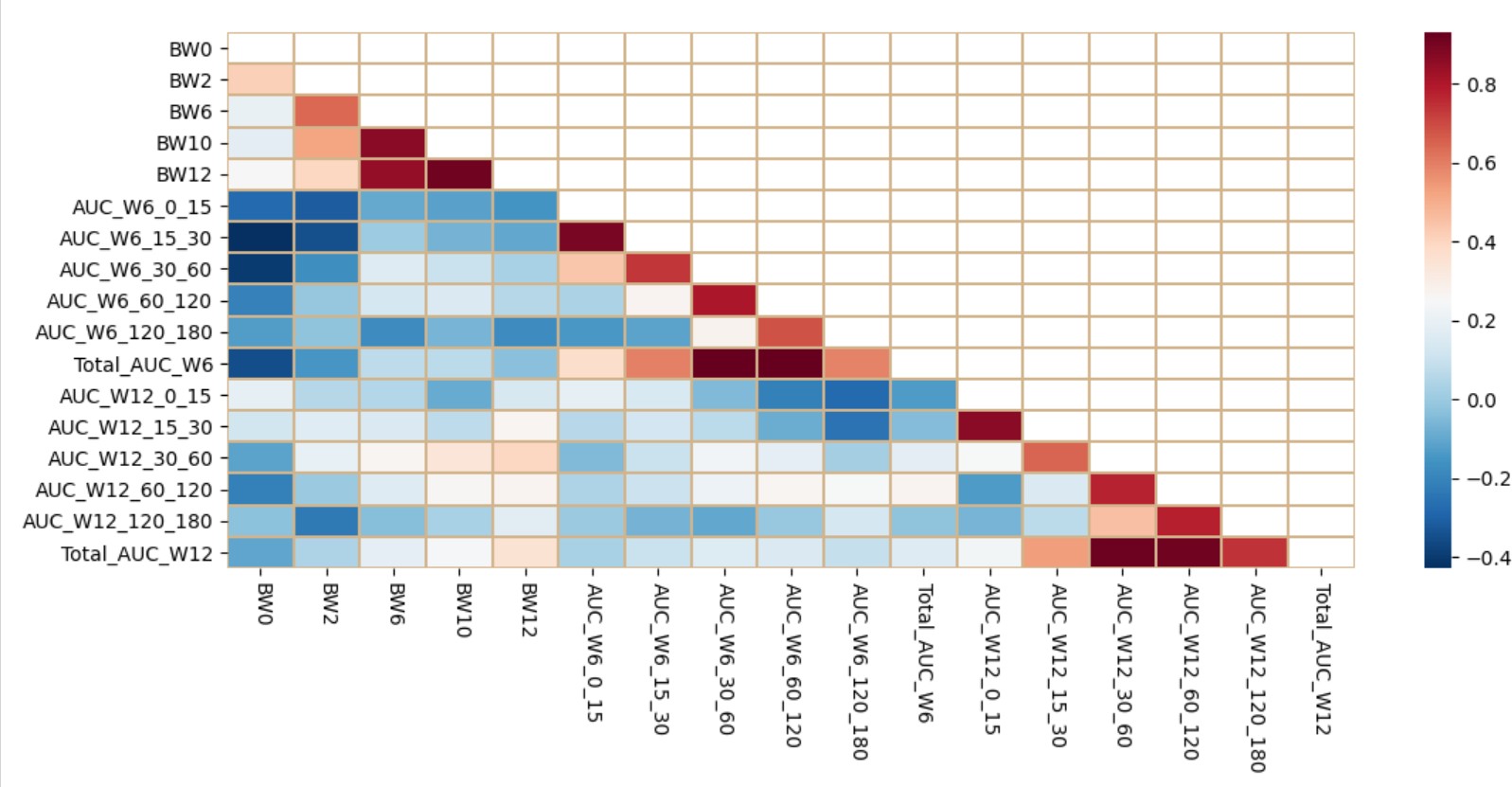


**Figure S18:** Heat map showing correlations between sex and individual CC line: BW and glucose tolerance referred here as AUC, at week 6 and week 12 of the experiment for females of line 1912 among the different conditions of the experiment. Each map presents the influence of BW on AUC. According to the color key, the correlation coefficient between -1≤ r ≤1 is significant at p<0.01 (**) and p<0.05 (*).

Line 1912 – M


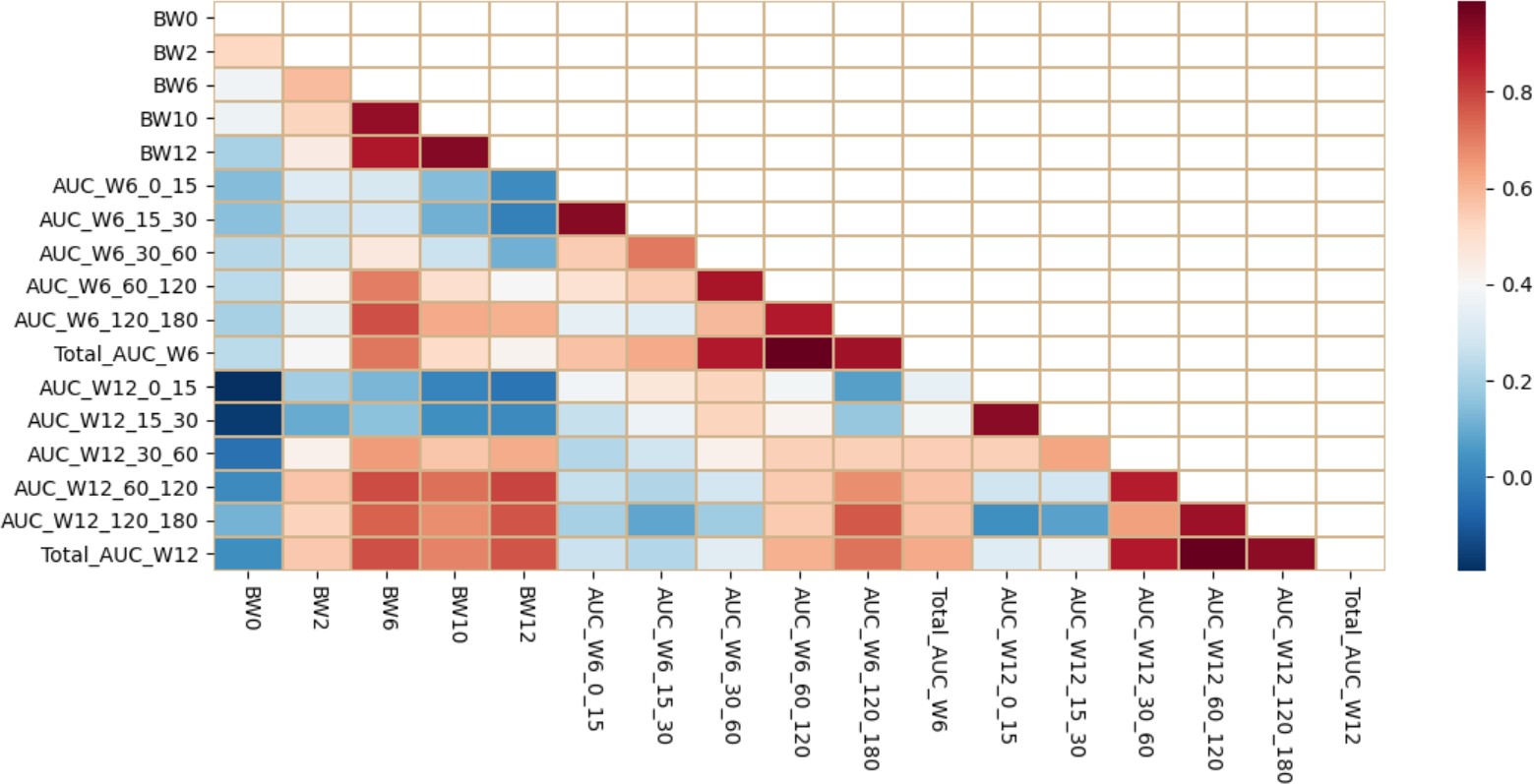


**Figure S19:** Heat map showing correlations between sex and individual CC line: BW and glucose tolerance referred here as AUC, at week 6 and week 12 of the experiment for males of line 1912 among the different conditions of the experiment. Each map presents the influence of BW on AUC. According to the color key, the correlation coefficient between -1≤ r ≤1 is significant at p<0.01 (**) and p<0.05 (*).

Line 3912 – F


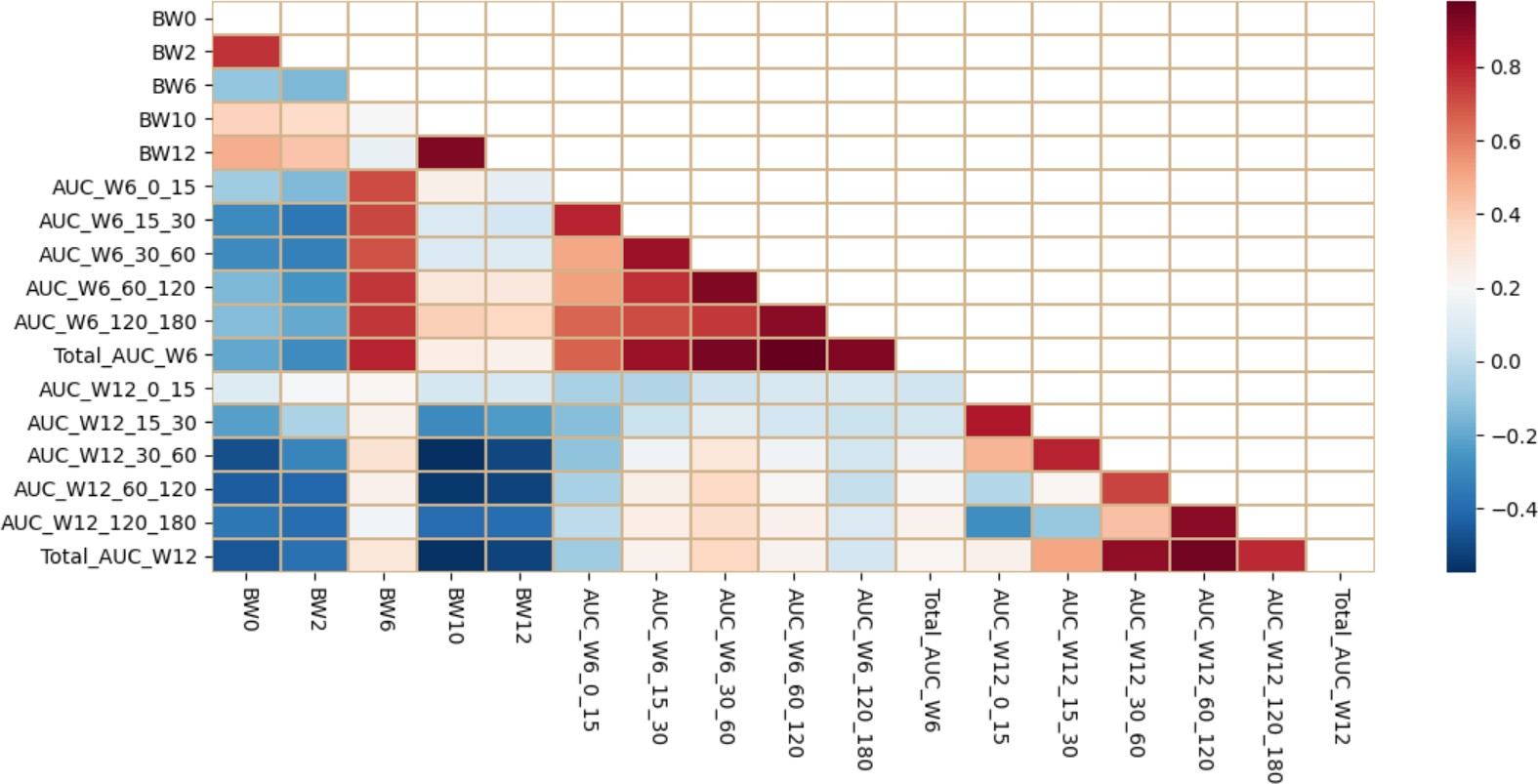


**Figure S20:** Heat map showing correlations between sex and individual CC line: BW and glucose tolerance referred here as AUC, at week 6 and week 12 of the experiment for females of line 3912 among the different conditions of the experiment. Each map presents the influence of BW on AUC. According to the color key, the correlation coefficient between -1≤ r ≤1 is significant at p<0.01 (**) and p<0.05 (*).

Line 3912 – M


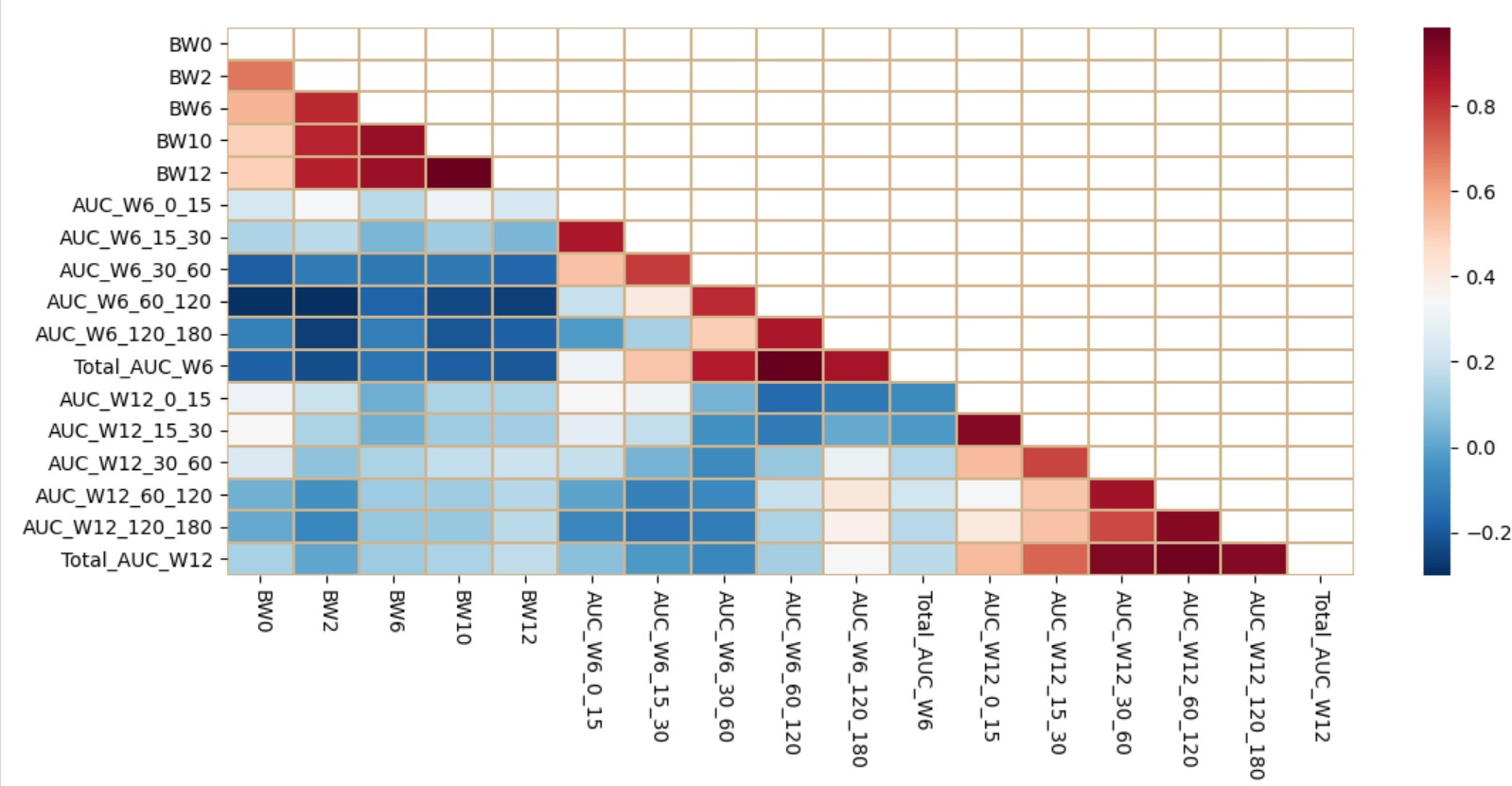


**Figure S21:** Heat map showing correlations between sex and individual CC line: BW and glucose tolerance referred here as AUC, at week 6 and week 12 of the experiment for males of line 3912 among the different conditions of the experiment. Each map presents the influence of BW on AUC. According to the color key, the correlation coefficient between -1≤ r ≤1 is significant at p<0.01 (**) and p<0.05 (*).

Line 4141 – F


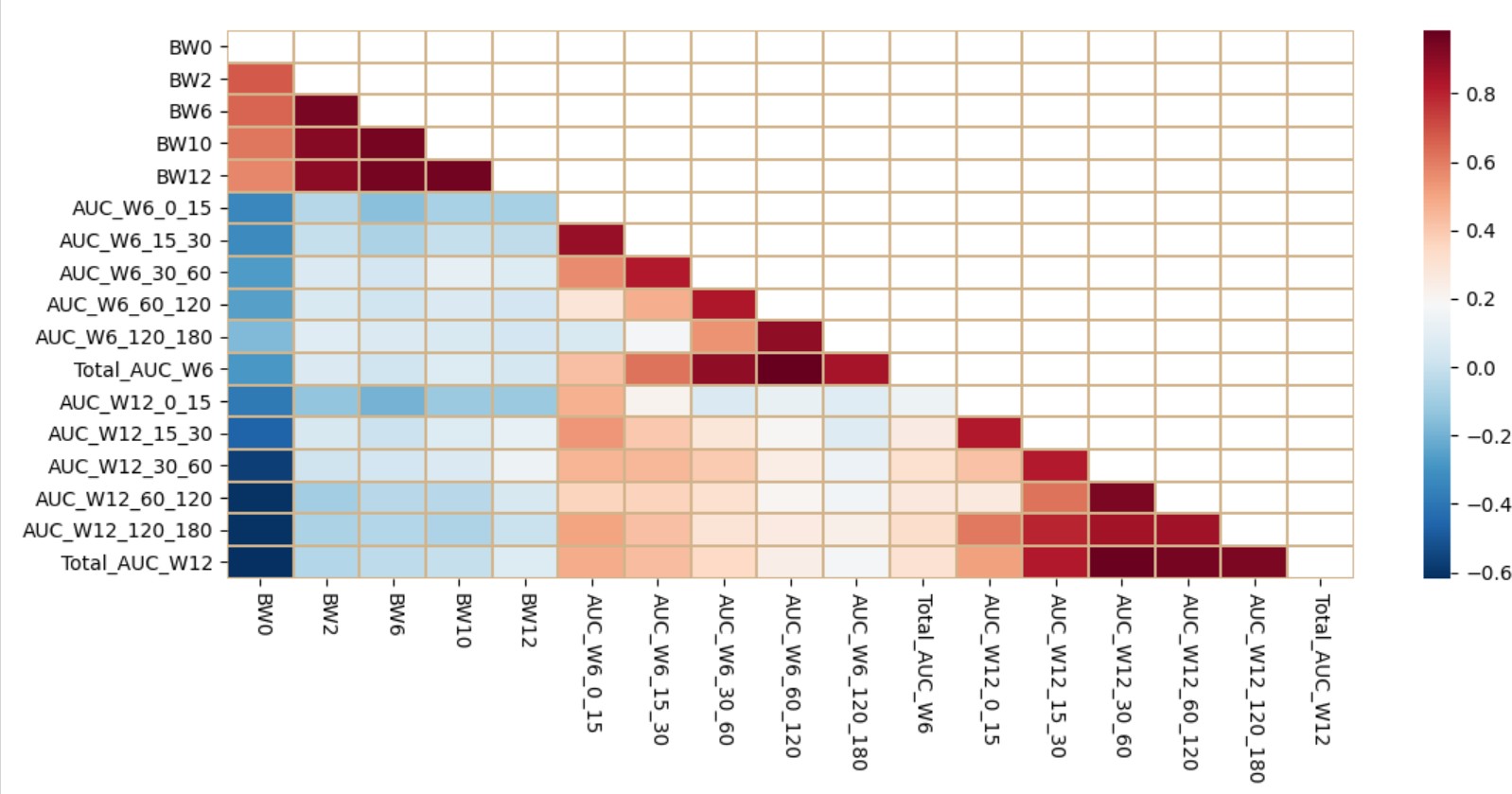


**Figure S22:** Heat map showing correlations between sex and individual CC line: BW and glucose tolerance referred here as AUC, at week 6 and week 12 of the experiment for females of line 4141 among the different conditions of the experiment. Each map presents the influence of BW on AUC. According to the color key, the correlation coefficient between -1≤ r ≤1 is significant at p<0.01 (**) and p<0.05 (*).

Line 4141 – M


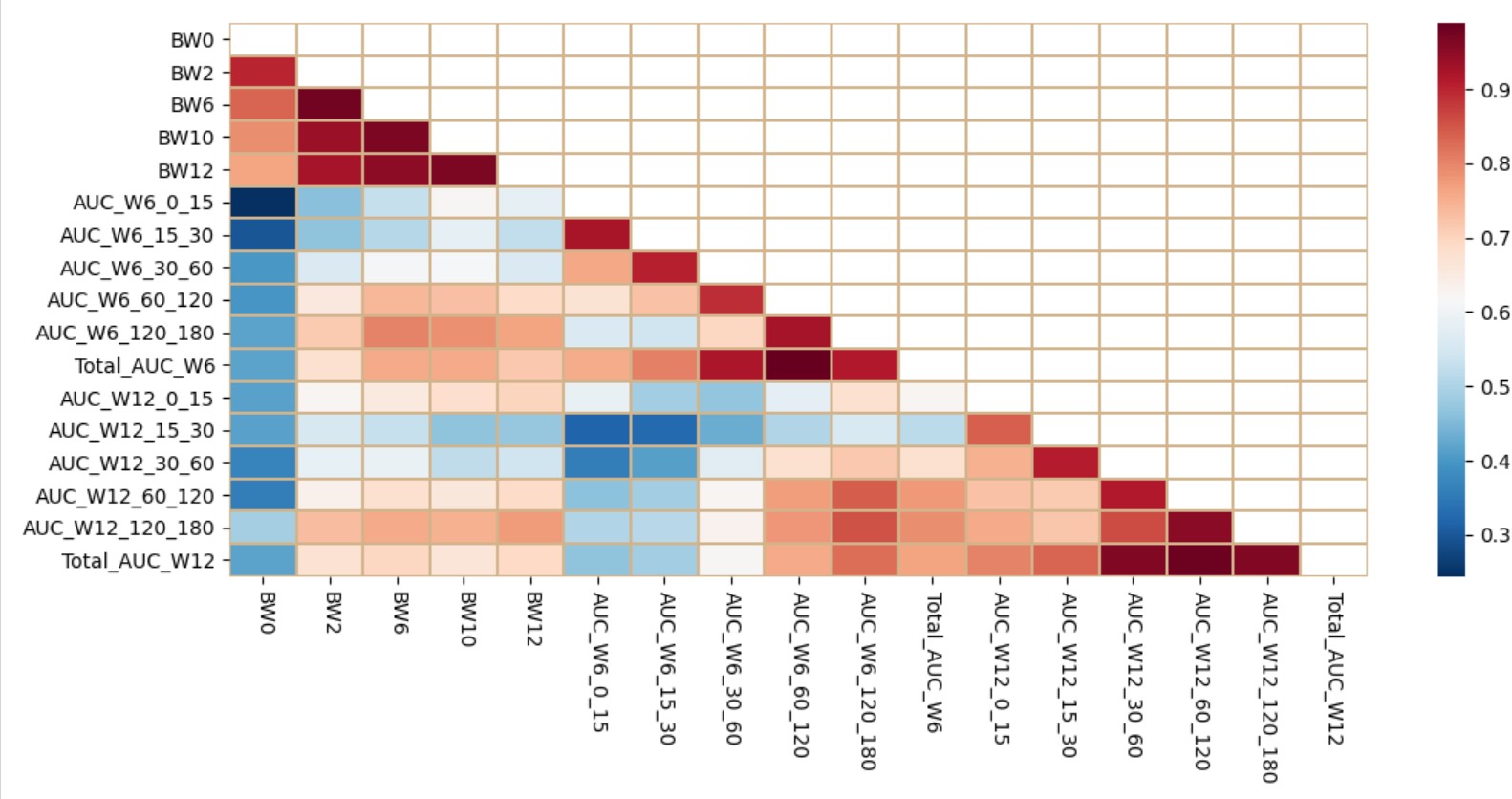


**Figure S23:** Heat map showing correlations between sex and individual CC line: BW and glucose tolerance referred here as AUC, at week 6 and week 12 of the experiment for males of line 4141 among the different conditions of the experiment. Each map presents the influence of BW on AUC. According to the color key, the correlation coefficient between -1≤ r ≤1 is significant at p<0.01 (**) and p<0.05 (*).

Line 6018 – F


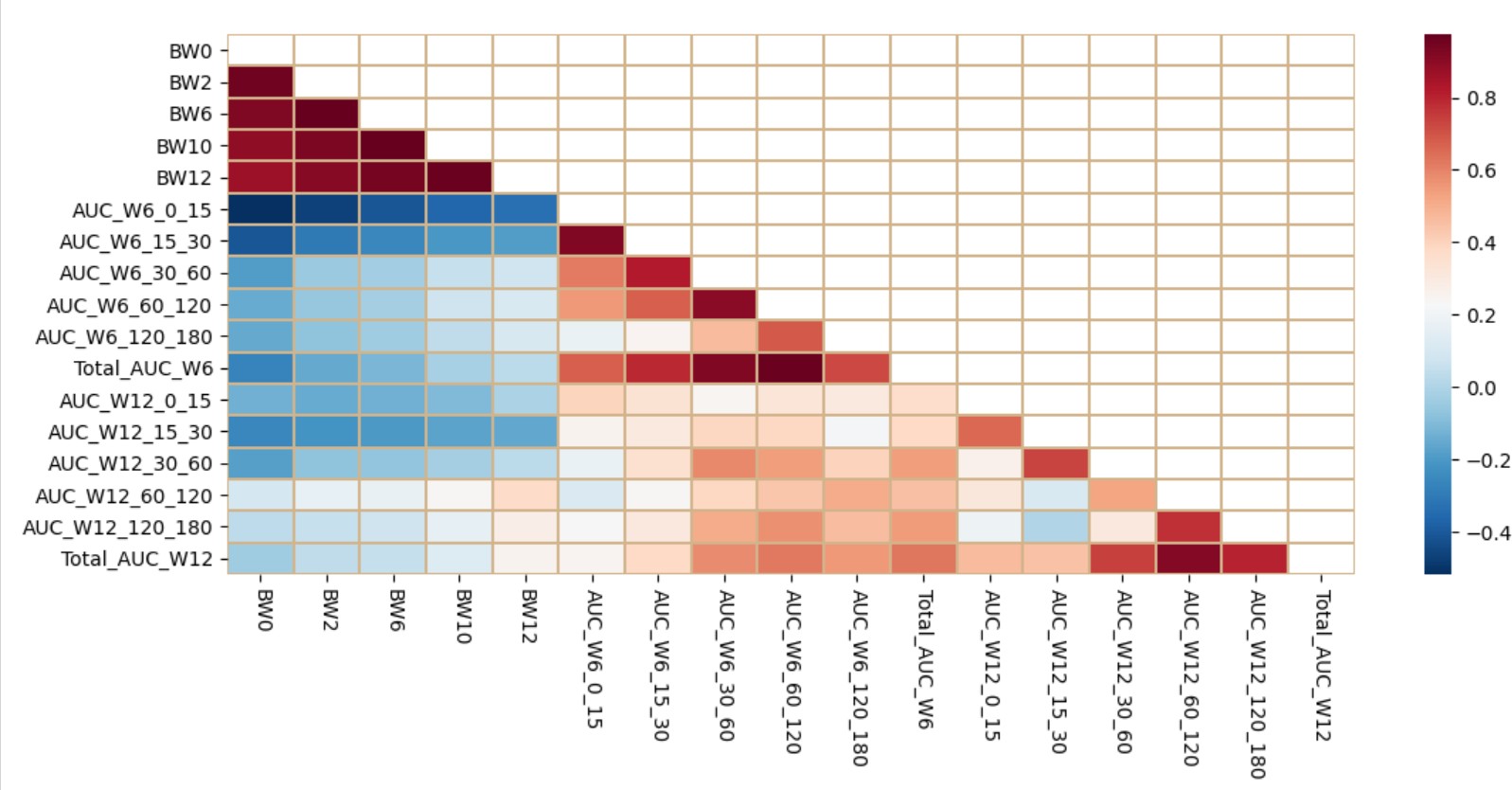


**Figure S24:** Heat map showing correlations between sex and individual CC line: BW and glucose tolerance referred here as AUC, at week 6 and week 12 of the experiment for females of line 6018 among the different conditions of the experiment. Each map presents the influence of BW on AUC. According to the color key, the correlation coefficient between -1≤ r ≤1 is significant at p<0.01 (**) and p<0.05 (*).

Line 6018 – M


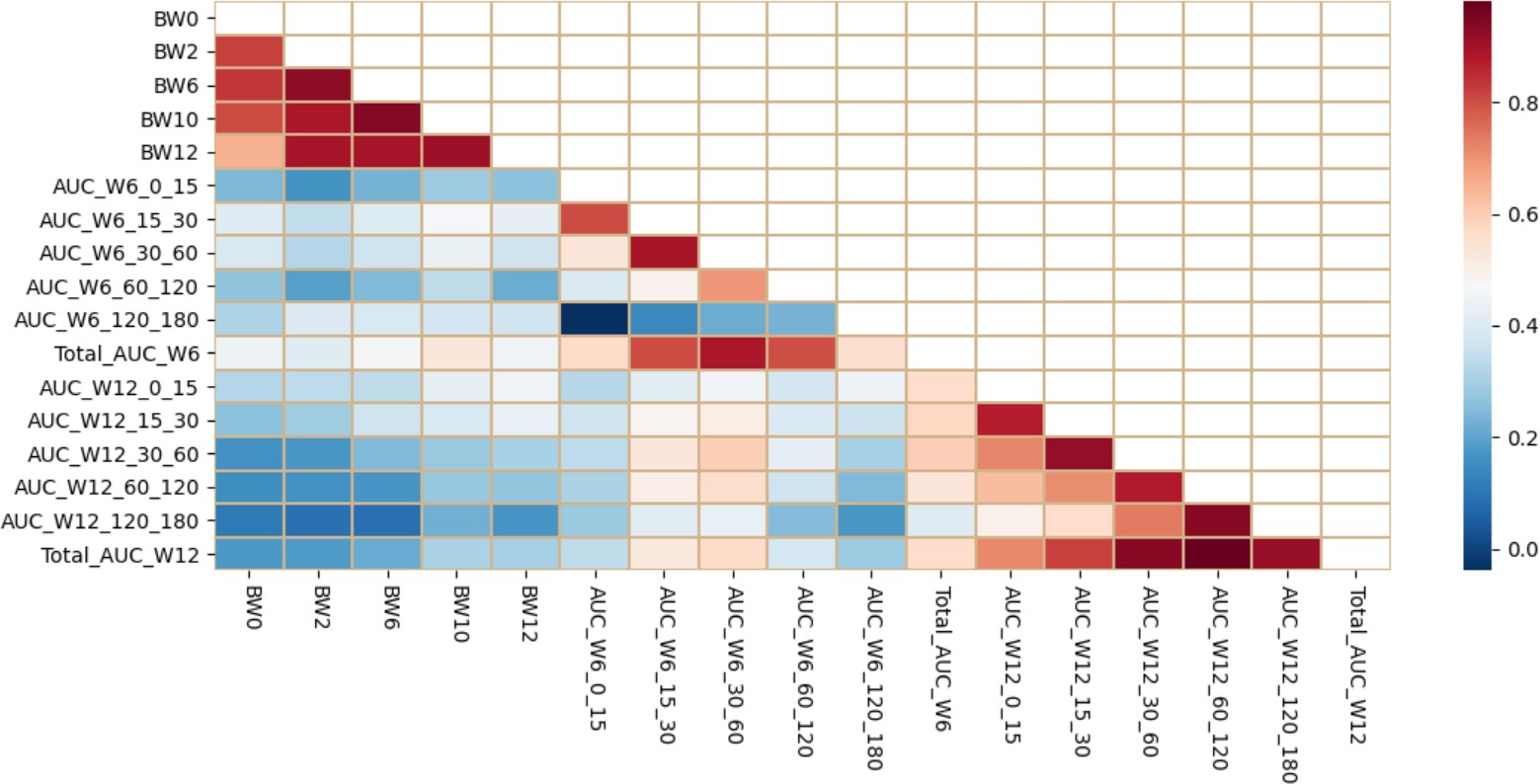


**Figure S25:** Heat map showing correlations between sex and individual CC line: BW and glucose tolerance referred here as AUC, at week 6 and week 12 of the experiment for males of line 6018 among the different conditions of the experiment. Each map presents the influence of BW on AUC. According to the color key, the correlation coefficient between -1≤ r ≤1 is significant at p<0.01 (**) and p<0.05 (*).

Line 5001 – F


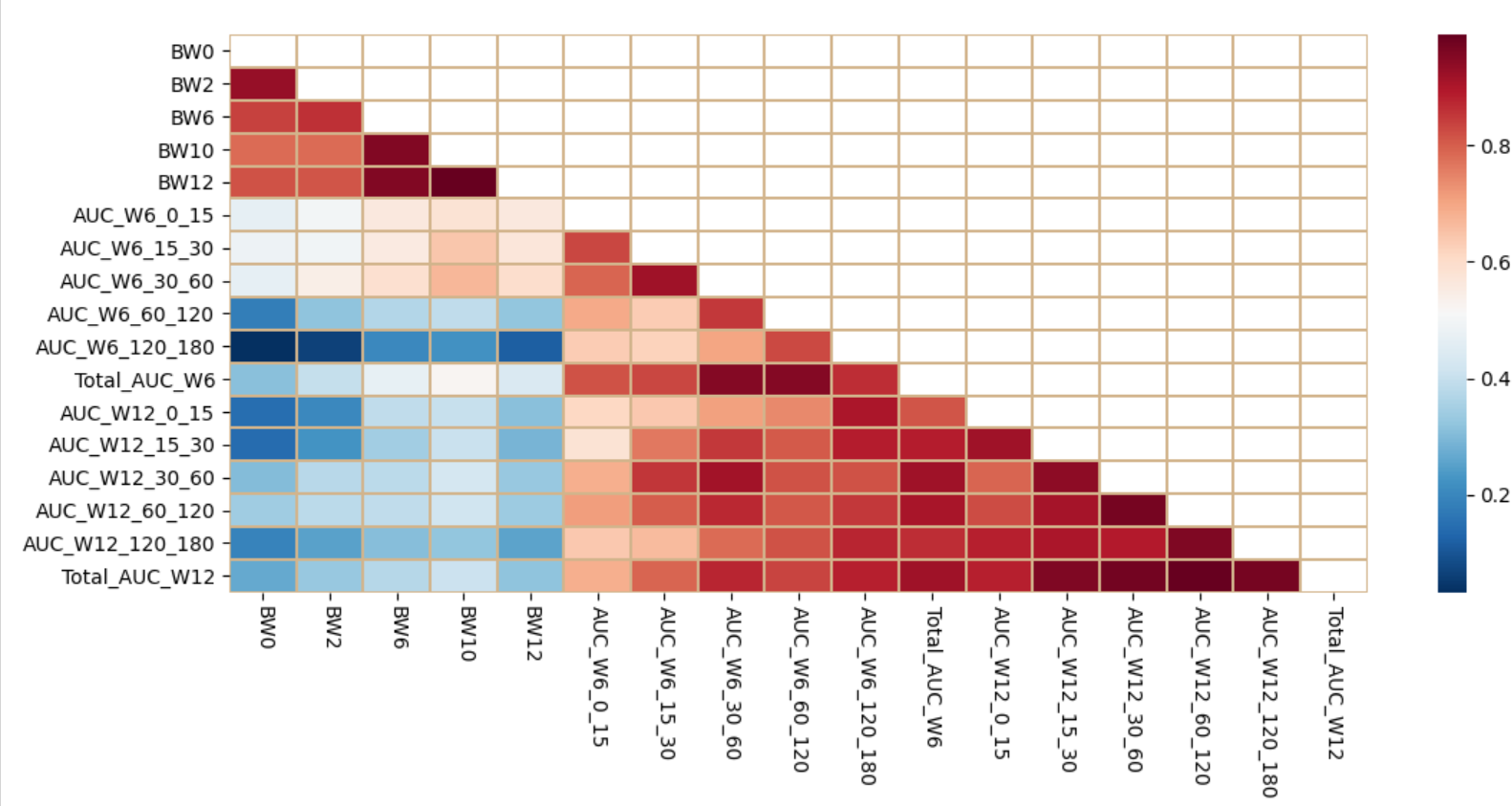


**Figure S26:** Heat map showing correlations between sex and individual CC line: BW and glucose tolerance referred here as AUC, at week 6 and week 12 of the experiment for females of line 5001 among the different conditions of the experiment. Each map presents the influence of BW on AUC. According to the color key, the correlation coefficient between -1≤ r ≤1 is significant at p<0.01 (**) and p<0.05 (*).

Line 5001 – M


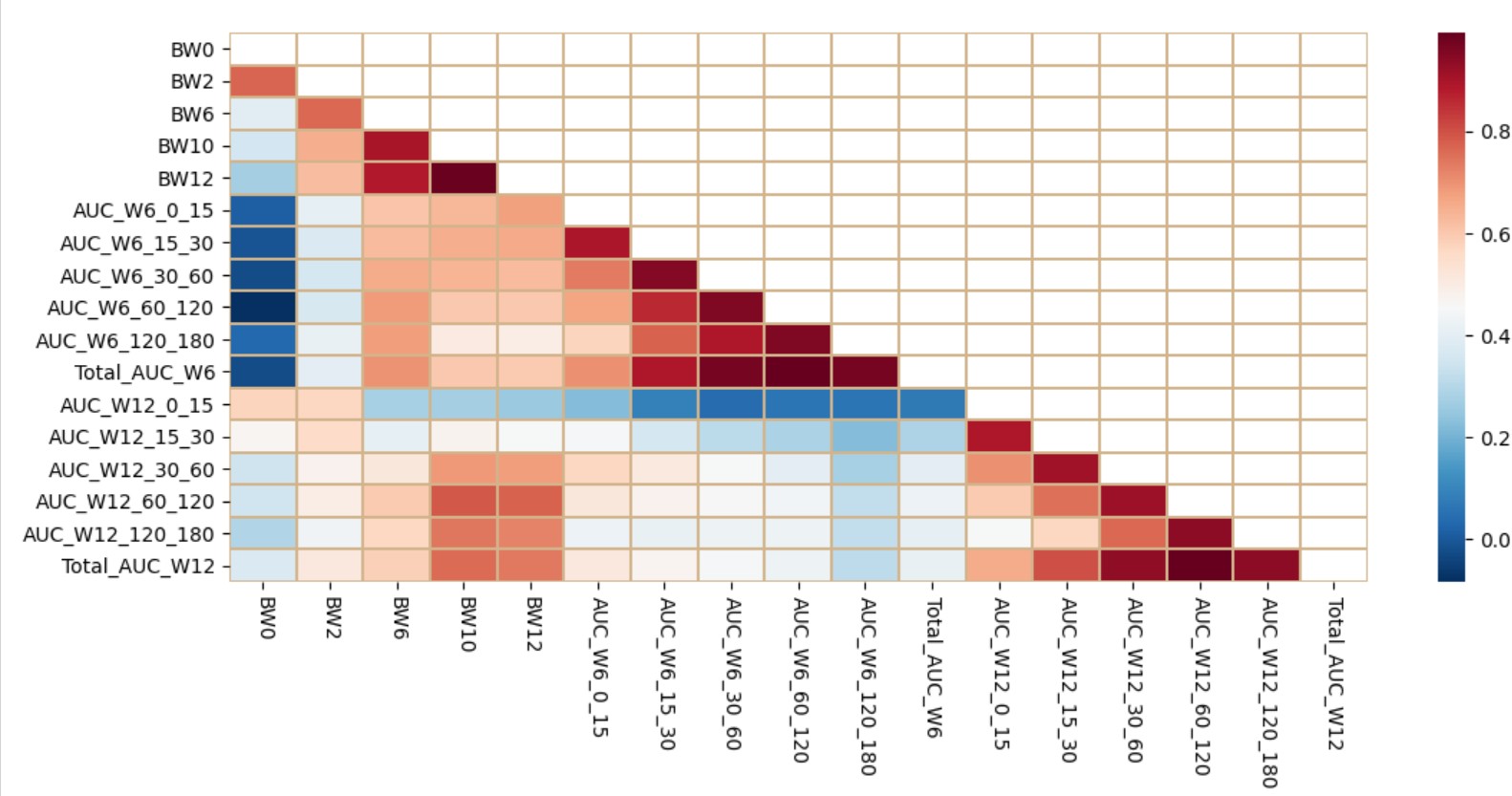


**Figure S27:** Heat map showing correlations between sex and individual CC line: BW and glucose tolerance referred here as AUC, at week 6 and week 12 of the experiment for males of line 5001 among the different conditions of the experiment. Each map presents the influence of BW on AUC. According to the color key, the correlation coefficient between -1≤ r ≤1 is significant at p<0.01 (**) and p<0.05 (*).

Line 3348 – F


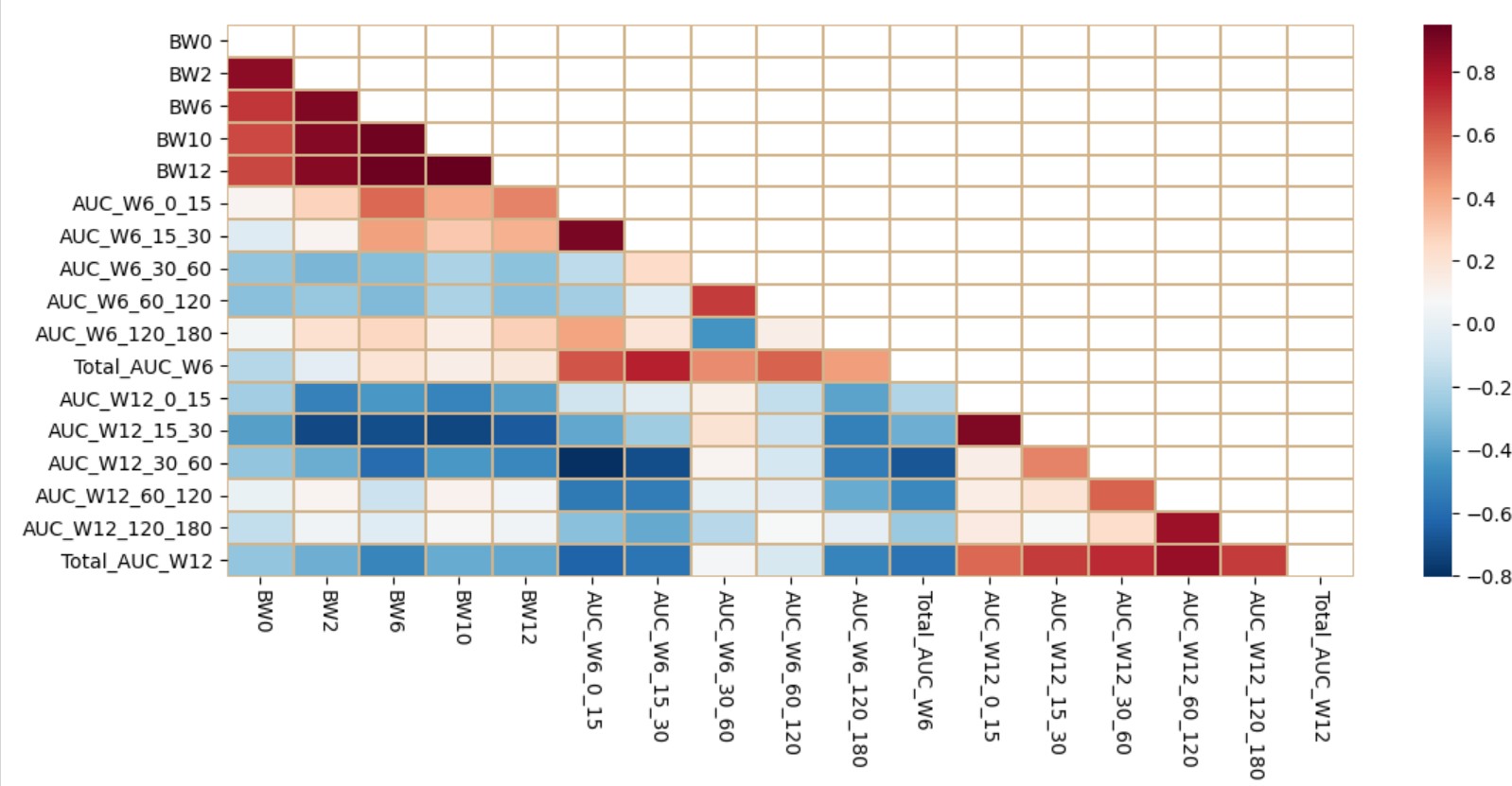


**Figure S28:** Heat map showing correlations between sex and individual CC line: BW and glucose tolerance referred here as AUC, at week 6 and week 12 of the experiment for females of line 3348 among the different conditions of the experiment. Each map presents the influence of BW on AUC. According to the color key, the correlation coefficient between -1≤ r ≤1 is significant at p<0.01 (**) and p<0.05 (*).

Line 3348 – M


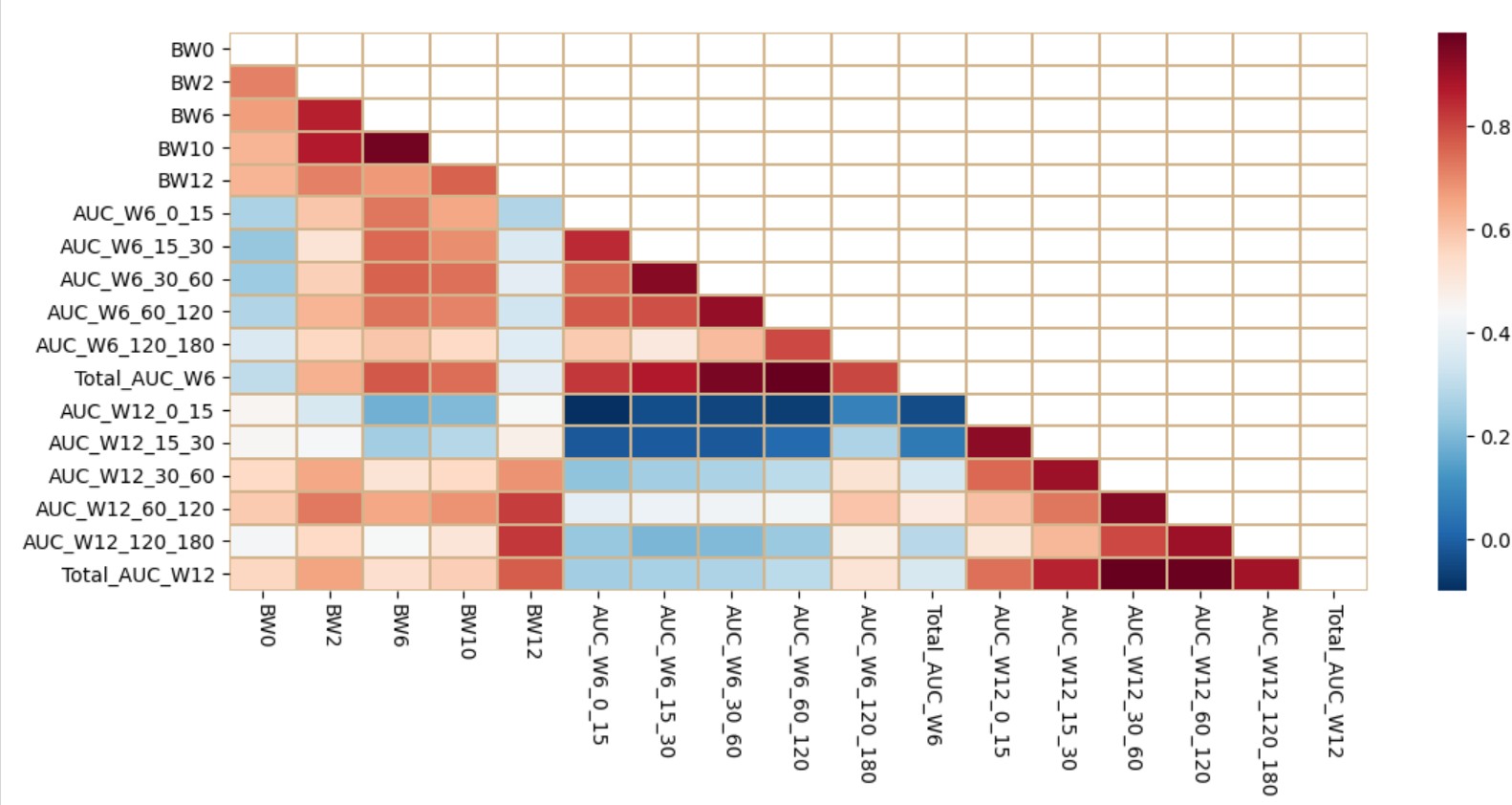


**Figure S29:** Heat map showing correlations between sex and individual CC line: BW and glucose tolerance referred here as AUC, at week 6 and week 12 of the experiment for males of line 3348 among the different conditions of the experiment. Each map presents the influence of BW on AUC. According to the color key, the correlation coefficient between -1≤ r ≤1 is significant at p<0.01 (**) and p<0.05 (*).

Line 111 – F


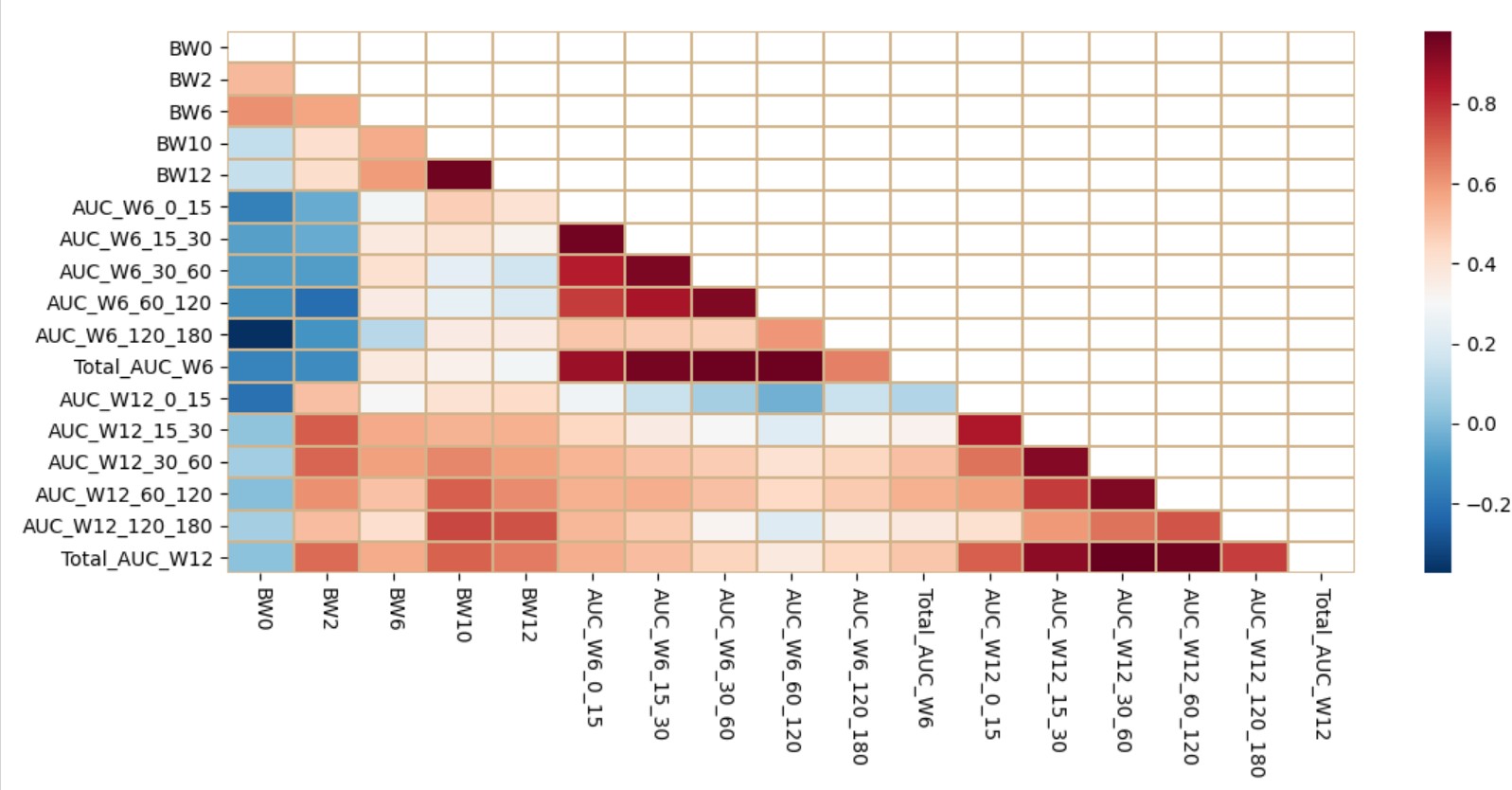


**Figure S30:** Heat map showing correlations between sex and individual CC line: BW and glucose tolerance referred here as AUC, at week 6 and week 12 of the experiment for females of line 111 among the different conditions of the experiment. Each map presents the influence of BW on AUC. According to the color key, the correlation coefficient between -1≤ r ≤1 is significant at p<0.01 (**) and p<0.05 (*).

Line 111 – M


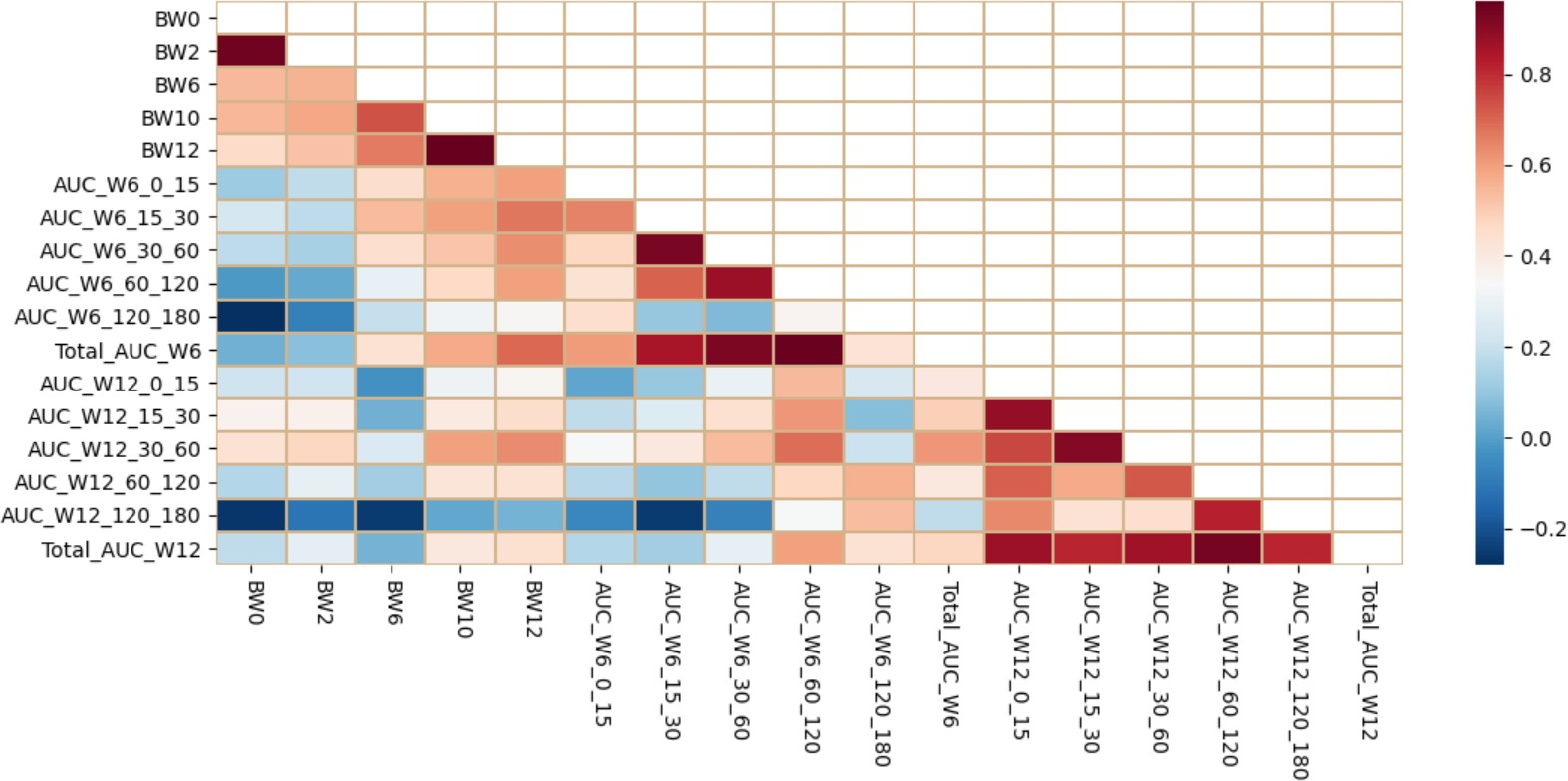


**Figure S31:** Heat map showing correlations between sex and individual CC line: BW and glucose tolerance referred here as AUC, at week 6 and week 12 of the experiment for males of line 111 among the different conditions of the experiment. Each map presents the influence of BW on AUC. According to the color key, the correlation coefficient between -1≤ r ≤1 is significant at p<0.01 (**) and p<0.05 (*).
